# MicroRNA as a Potential Diagnostic and Prognostic Biomarker in Diffuse Large B‐Cell Lymphoma: A Systematic Review and Meta‐Analysis

**DOI:** 10.1002/cnr2.70070

**Published:** 2025-01-24

**Authors:** Shaghayegh Khanmohammadi, Mahdi Masrour, Parisa Fallahtafti, Fatemeh Hasani

**Affiliations:** ^1^ School of Medicine Tehran University of Medical Sciences Tehran Iran; ^2^ Research Center for Immunodeficiencies, Pediatrics Center of Excellence, Children's Medical Center Tehran University of Medical Sciences Tehran Iran; ^3^ Non‐Communicable Diseases Research Center, Endocrinology and Metabolism Population Sciences Institute Tehran University of Medical Sciences Tehran Iran; ^4^ Tehran Heart Center, Cardiovascular Diseases Research Institute Tehran University of Medical Sciences Tehran Iran; ^5^ Golestan Research Center of Gastroenterology and Hepatology Golestan University of Medical Sciences Gorgan Iran

**Keywords:** biomarker, diagnosis, diffuse large B‐cell lymphoma, microRNA, prognosis

## Abstract

**Background:**

Recently, microRNAs (miRNAs) have been applied as biomarkers for diffuse large B‐cell lymphoma (DLBCL) patients. Early diagnosis and management of DLBCL can improve patient survival and prognosis.

**Aims:**

This systematic review and meta‐analysis aimed to evaluate the diagnostic and prognostic accuracy of miRNA biomarkers in DLBCL patients.

**Methods:**

We used the keywords “diffuse large B‐cell lymphoma” and “microRNA” to search databases for original publications until June 14, 2023. Specificity, sensitivity, and AUC were used to assess diagnostic accuracy, and the prognostic value was assessed using the overall survival (OS) and progression‐free survival (PFS) hazard ratio (HR). A subgroup analysis was performed based on the sample type acquired to investigate the heterogeneity.

**Results:**

Thirteen diagnostic and 33 prognostic studies were included from 839 articles. The Reitsma bivariate model estimated a sensitivity of 0.788 (95% CI: 0.733–0.834, *p <* 0.001), a specificity of 0.727 (95% CI: 0.654–0.790, *p <* 0.001), and an AUC of 0.824 in. The pooled AUC was 0.7385 (95% CI: 0.6847–0.7923, *p* < 0.0001). The pooled OS and PFS HRs (> 1) were 2.2847 (95% CI: 1.7248–3.0263, *p* < 0.0001) and 2.4883 (95% CI: 1.7367–3.5650, *p* < 0.0001). The pooled OS and PFS HRs (< 1) were 0.4965 (95% CI: 0.3576–0.6894, *p* < 0.0001) and 2.4883 (95% CI: 1.7367–3.5650, *p* < 0.0001). MiR‐155 diagnostic values had a sensitivity of 0.710 (*p* > 0.1) and a specificity of 0.725 (*p* < 0.05), with an AUC of 0.776. miR‐21 diagnostic values had an AUC of 0.8468 (*p* < 0.0001) and OS HR of 2.8938.

**Conclusion:**

MicroRNAs could serve as a powerful diagnostic and prognostic tool in DLBCL.

## 1 Introduction

Diffuse large B‐cell lymphoma (DLBCL) is the prevailing form of lymphoma and represents around 30% of all non‐Hodgkin lymphoma cases, with roughly 25 000 new cases each year in the United States and an estimated incidence rate of 4.68 cases per 100 000 persons each year [1]. The diagnosis of DLBCL primarily relies on invasive risk‐posing procedures like analyzing biopsy tissue [2, 3]. Furthermore, despite the widespread use of the international prognostic index (IPI) as a prognostic factor for DLBCL cases [4], the existing prognostic evaluation system demonstrates limitations, as patients categorized into similar prognostic groups frequently demonstrate divergent clinical outcomes [5]. Early diagnosis and subsequent management of recurrent lymphoma can significantly contribute to improving patient survival and prognosis [6]. Thus, there is a need for biomarkers that can aid in the diagnosis and prognosis of DLBCL.

Micro ribonucleic acids (miRNAs), classified as noncoding RNAs with a size range of 21–23 nucleotides, actively participate in the modulation of gene expression by engaging in interactions with miRNA molecules at the posttranscriptional level [7]. Multiple investigations have indicated that miRNAs derived from peripheral blood hold promise as non‐invasive biomarkers capable of detecting tumors, monitoring disease progression, and assessing the effectiveness of therapeutic interventions [8, 9, 10]. Numerous studies have elucidated the pivotal involvement of specific miRNAs in cancer progression [11]. Therefore, establishing the assessment of miRNA as a promising avenue for identifying biomarkers in the early diagnosis, monitoring, and detection of cancer patients can be a new insight [12].

Recent studies have investigated the role of non‐coding RNAs as prognostic and diagnostic biomarkers in various diseases [13, 14, 15]. For instance, Khare et al. utilized small‐RNA sequencing to observe altered plasma levels of specific miRNAs in DLBCL. They found increased levels of miRNAs, including miR‐425, miR532‐5p, and miR‐124, while miR‐345 and miR‐197 were found to be decreased [6]. The results of another study on miRNAs highlighted the potential of miR‐155 in extracellular vesicles (EVs) as a promising diagnostic tool in hematologic malignancies [16]. Another study indicated that elevated levels of miR‐125b were associated with an unfavorable prognosis, and patients with higher miR‐125b levels exhibited shorter overall survival (OS) in DLBCL; the results suggested that monitoring miR‐125b and miR‐130a in the blood could be a valuable method for predicting how DLBCL patients will respond to treatment and assessing their disease status [17].

miRNAs exhibit enhanced stability due to their protective lipid bilayer membrane, differentiating them from other noninvasive biomarkers [18, 19]. Furthermore, the diagnostic and prognostic implications of other non‐invasive biomarkers, such as plasma exosomal nucleic acid (exoNA), exosomal DNA, plasma circulating free DNA (cfDNA), and exosomal RNA, are potentially complicated as they are released passively by necrotic and apoptotic cells [20]. Our systematic and meta‐analysis study aimed to comprehensively evaluate the diagnostic and prognostic accuracy of miRNA biomarkers in patients with DLBCL. We conducted a thorough analysis and integrated current research studies to offer valuable insights into the potential of miRNAs as reliable and informative markers for diagnosing and predicting the prognosis of DLBCL. Our findings could contribute to the advancement of improved patient outcomes in the management of DLBCL.

## 2 Methods

This systematic review and meta‐analysis followed PRISMA items [21]. Our study protocol was registered at PROSPERO under the registration number CRD42023441555.

### 2.1 Literature Search

On June 14th, 2023, a comprehensive search of the literature was done in PubMed, Web of Science (ISI), Scopus, and Embase for English publications, without constraints on publication year the following medical subject headings (MeSH) terms and free keywords: “Diffuse large B‐cell lymphoma” and “microRNA.” The search query is provided in Supporting Information.

### 2.2 Selection Criteria

This study included peer‐reviewed original research that presented sensitivity, specificity, or AUC values of microRNAs in the diagnosis of DLBCL, as well as their association with OS, progression‐free survival (PFS), disease‐free survival (DFS), recurrent‐free survival (RFS), and event‐free survival (EFS). The diagnostic section of our study included original case–control human studies, while the prognostic section employed cohort studies. These studies were conducted either prospectively or retrospectively, using samples obtained from individuals pathologically diagnosed with cancer and healthy participants. Regardless of assay duration, diagnostic accuracy studies should have compared microRNAs to an adequate reference control to assess sensitivity, specificity, and AUC. Eligibility restrictions were not applied based on the healthcare settings in which the research was conducted, nor were they based on the total number of participants in the included studies. Studies that were not peer‐reviewed, conducted in languages other than English, and utilized datasets or animal models, letters, commentaries, reviews, case reports, and case series were considered ineligible and, therefore, excluded from the analysis.

After deleting duplicates, S.K. and P.F. assessed the titles and abstracts of all identified papers for eligibility using the stated inclusion and exclusion criteria. Upon gathering studies that met the criteria for eligibility, both authors proceeded to independently conduct a thorough review of the full texts. Any conflicts that emerged during the review process were successfully resolved through the establishment of a consensus.

### 2.3 Data Extraction

The data were extracted from the included studies by two reviewers (S.K. and M.M.) independently using a dedicated electronic spreadsheet. The collected information included author, publication year, study design, specimen type, control population, sample size, miRNA name, changes in miRNA levels in patients compared with the control group, diagnostic or prognostic performance measures (such as sensitivity, specificity, AUC with corresponding 95% confidence interval [CI] and *p* value), as well as mean, median, and hazard ratio (HR) for survival outcomes with corresponding 95% CI and *p* value. Any discrepancies between reviewers were resolved through discussion and consensus.

### 2.4 Quality Assessment

We used the Newcastle–Ottawa Scale (NOS) for cohort and case–control studies to assess the quality of included studies [22]. Two authors (F.H. and S.K.) independently assessed the quality based on NOS criteria. Any disagreements in the quality assessment were resolved through discussion. The NOS evaluates studies based on three main categories of bias: selection, comparability, and outcome. Scores ranging from 7 and above, 2 to 6, and 1 and below were classified as “good,” “fair,” and “poor,” respectively.

### 2.5 Statistical Analysis

To pool research providing diagnostic specificity and sensitivity, we utilized Reitsma et al.'s bivariate random effect model [23]. Considering their interdependency, a bivariate model employs logit transformation to aggregate test sensitivity and specificity across studies. The summary receiver operating characteristic (sROC) curve and AUC, which represent test precision, are also determined in this model. For the studies that reported AUCs, the random effects model was utilized to meta‐analyze diagnostic values using the inverse variance method. Because of the expected variability across the included research, the random effect was adopted.

The inverse variance method with logarithmic HR values was utilized for the meta‐analysis of prognostic values presented with HRs. The random effect model was employed to compensate for the heterogeneity seen among the reported values. Since HR less than one indicates a cancer‐protective function of the studied variable (microRNAs) and HR more than one indicates a cancer‐promoting role of the variable, these two groups were separated as different categories independently of the regulation of the studied microRNA.

The standard error of the AUCs and HRs for use in the meta‐analysis was determined using the 95% CI if provided or the AUC value itself and the sample size. To examine research heterogeneity, *I* ^2^ and DerSimonian–Laird estimator of *τ* ^2^ statistics were utilized. A subgroup analysis was performed depending on the kind of samples acquired to further investigate the heterogeneity. R version 4.2.2 was used for the statistical analysis and visualizations. An *I* ^2^ value of more than 50% and a *p* value less than 0.05 were considered statistically significant.

## 3 Results

### 3.1 Basic Characteristics

**FIGURE 1:**
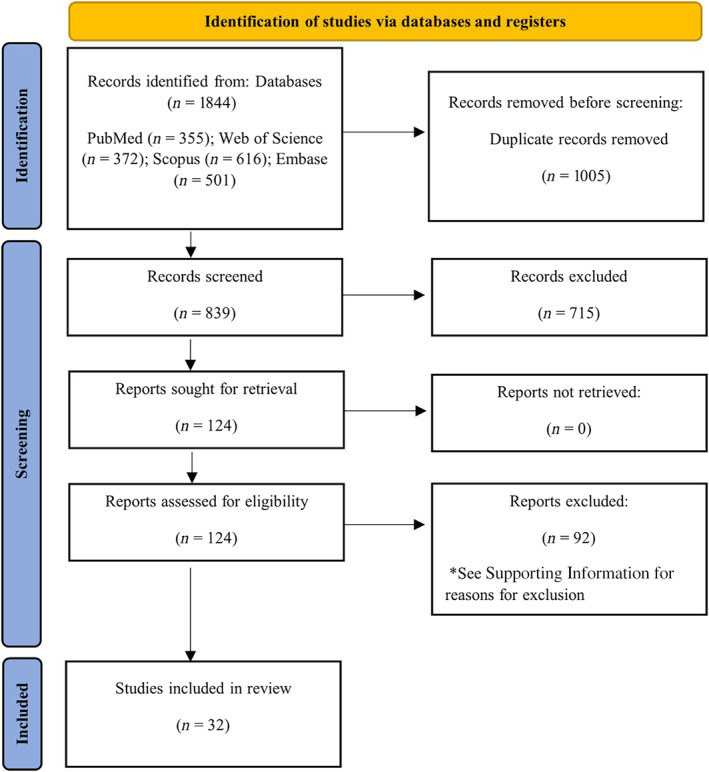
The PRISMA flowchart.

Following the initial examination of the database, a total of 1844 titles were obtained. Following the removal of duplicate articles, 839 articles were evaluated for inclusion. Following a thorough examination of titles and abstracts, 715 publications were ineligible for full‐text review, while 124 were deemed suitable. Thirteen studies with 44 diagnostic evaluations met the inclusion criteria for the diagnostic section, while 33 studies with 73 prognostic evaluations met the inclusion criteria for the section on prognosis (total = 32). The excluded investigations are detailed in Supporting Information. All of the 44 diagnostic evaluations were used in the meta‐analysis, while only 28 prognostic evaluations were used in the meta‐analysis. Figure 1 depicts the PRISMA flowchart, which illustrates the process of selecting and excluding studies.

Table 1 provides a concise summary of the primary characteristics of the included studies. The papers in the diagnosis segment were published between 2012 and 2022, whereas those in the prognosis section were published between 2007 and 2022. The diagnosis segment included 2475 DLBCL cases and 1703 healthy controls from Poland, the United States, Egypt, China, Denmark, and Israel. To assess OS, the prognosis section analyzed 2185 DLBCL cases from China, Korea, Iran, Spain, and Italy. In addition, the PFS of 1583 cases from China, Korea, and Italy was evaluated. The 44 diagnostic evaluations involved two categories of specimens: eight tumor tissue samples and 36 blood samples. The prognostic evaluations also involved two specimen categories: tumor tissue and blood samples.

**TABLE 1:** Basic characteristics of the included studies.

Prognosis section																			
ID	Author, year	Country	DLBCL type	Specimen	Control type	Case number	Control number	miRNA	Up/downregulation	Overall survival (95% CI)	*p*	Progression‐free survival (95% CI)	*p*	Disease‐free survival (95% CI)	*p*	Recurrent‐free survival (95% CI)	*p*	Event‐free survival (95% CI)	*p*
1	Abou Elnour, 2022 [24]	Egypt	DLBCL	Blood	Healthy control	50	50	miR‐150	Down	Mean low: 17.200 (16.13–18.27)	0.258								
								miR‐150	Down	High: 16.440 (15.21–17.67)	0.258								
								miR‐21	Up	Low: 18 (18–18)	0.005								
								miR‐21	Up	High: 15.64 (14.13–17.15)	0.005								
2	Ahmadvand, 2018 [25]	Iran	DLBCL	Plasma	Healthy control	40	38	miR‐155	Up	Median high: 9 (NA–NA)	0.043								
								miR‐155	Up	Low: 13 (NA–NA)	0.043								
								miR‐155	Up	HR: 2.7 (0.84–16.3)	0.049								
3	Alencar, 2011 [26]	Canada, Spain, USA	DLBCL	Tissue	—	176	—	miR‐18a	Up^a^	Coefficient: 0.752	0.011	Coefficient: 0.255							
								miR‐181a	Down^a^	Coefficient: −3.603	0.004	Coefficient: −3.207	0.005						
								MiR‐222	Up^a^	Coefficient: 0.363	NS	Coefficient: 0.817	0.008						
4	Bedewy, 2017 [27]	Egypt	DLBCL	Serum	Healthy control	54	15	miR‐155	Up									OR: 5.916 (1.134–30.864)	0.035
5	Bento, 2022 [28]	Spain	DLBCL	Tissue	—	96	—	miR‐1244	Up	y7 low: 77% (64–90) High: 53% (30–76)	0.024							7y low: 72% (58–87) High: 32% (9–56)	0.005
							
miR‐193b‐5p	Up	7y low: 84% (72–97) High: 51% (30–71)	0.009							7y low: 79% (64–93) High: 34% (14–53)	< 0.001
							
miR‐1231	Up	HR: 4.4 (1.6–12.2)	0.004							HR: 5.6 (2.3–13.6)	< 0.001
							
miR‐1231	Up	7y low: 80% (67–93) High: 51% (28–73)	0.02							7y low: 75% (60–89) High: 29% (2–55)	0.001
							
miR‐20b‐5p	Up	7y low: 78% (61–95) High: 63% (46–79)	0.21							7y low: 73% (54–92) High: 49% (32–65)	0.046
							
miR‐17‐3p	Up	7y low: 57% (20–94) High: 70% (57–83)	0.35							7y low: 21% (0–56) High: 62% (48–76)	0.053
							
miR‐182‐5p	Up	7y low: 92% (78–100) High: 61% (47–76)	0.043							7y low: 52% (34–70) High: 62% (42–82)	0.12
							
miR‐199a‐5	Up	7y low: 81% (67–95) High: 55% (36–74)	0.035							7y low: 69% (53–86) High: 45% (26–64)	0.04
							
miR‐6840‐3p	Up	7y low: 69% (55–84) High: 67% (45–89)	0.72							7y low: 68% (53–82) High: 33% (9–57)	0.01
							
miR‐885‐3p	Up	7y low: 79% (65–94) High: 52% (32–73)	0.008							7y low: 70% (45–95) High: 52% (36–67)	0.12
							
miR‐6806‐5p	Up	7y low: 72% (56–87) High: 65% (45–84)	0.43							7y low: 69% (54–85) High: 42% (20–63)	0.019
6	Berglund, 2012 [29]	Sweden	DLBCL	Tissue	Healthy control	61	13	miR‐200c	Up^a^	Median high: 20.3	0.019								
								miR‐200c	Up^a^	Low: 35.8	0.019								
7	Cao, 2022 [30]	China	DLBCL	Blood	Healthy control	123	89	miR‐451a	Down	HR: 0.331 (0.159–0.69)	0.03	HR: 0.444 (0.243–0.813)	0.009						
8	Chen, 2020 [31]	China	PGI‐DLBCL	Tissue	Reactive LN	80	46	miR‐130a	Up	HR: 2.516 (1.046–6.052)	0.039	HR: 2.828 (1.143–6.994)	0.024						
9	Chen, 2021 [32]	China	DLBCL	Tissue	Reactive LN	80	80	miR‐383‐5p	Down	HR: 0.844 (0.754–0.945)	0.003			HR: 0.753 (0.672–0.842)	< 0.001				
10	Go, 2015 [33]	Korea	DLBCL	Tissue	Healthy control	200	11	miR‐21	Up	HR: 2.1 (1.1–3.9)	0.02	HR: 2.3 (1.1–4.8)	0.032						
								miR‐17‐92	Up			HR: 2.2	0.023						
11	He, 2014 [34]	China	DLBCL	Tissue	Healthy control	58	5	miR‐34a	Down	Median negative: 6.3	0.009	Negative: 11.7	0.024						
								miR‐34a	Down	Positive: 28.6	0.009	Positive: 33.9	0.024						
12	Hedström, 2013 [35]	Sweden	DLBCL	Tissue	Healthy control	59	13	miR‐129‐5p	Down	Median low: 23	0.0042								
								miR‐129‐5p	Down	High: 58	0.0042								
13	Hu, 2021 [36]	China	DLBCL	Tissue	Reactive LN	95	8	miR‐208a‐5p	Down^a^	HR: 0.626 (0.285–1.385)	0.249								
								miR‐296‐5p	Down^a^	HR: 0.398 (0.184–0.863)	0.02								
								miR‐1304‐5p	Up^a^	HR: 5.259 (2.364–11.7)	0.001								
14	Huang, 2022 [37]	China	DLBCL	Serum	—	72		miR‐146a	Down^a^	HR: 0.412 (0.139–1.22)	0.12	HR: 0.362 (0.135–0.97)	0.048						
15	Ji, 2022 [38]	China	PGI‐DLBCL	Serum	Healthy control	156	52	miR‐21	Up	Median high: 35	< 0.01	Median high: 30	< 0.01						
								miR‐21	Up	Low: 48	< 0.01	Low: 42	< 0.01						
								miR‐21	Up	HR: 3.311 (1.123–4.343)	0.016	HR: 4.345 (2.123–8.123)	0.017						
16	Jia, 2018 [39]	China	DLBCL	Tissue	Reactive LN	202	10	miR‐27b	Down	HR: 0.33 (0.2–0.54)	< 0.001								
17	Lawrie, 2007 [40]	UK	DLBCL	Tissue	Healthy control	35	6	miR‐21	Up							HR: 0.77 (0.61–0.97)	0.025		
18	Li, 2015 [41]	China	DLBCL	Serum	Healthy control	112	45	miR‐21	Up	HR: 4.404 (1.77–10.956)	0.001								
19	Marchesi, 2018 [42]	Italy	DLBCL	Serum	Healthy control	36		miR‐22	Down	HR: 5.19 (1.38–19.56)	0.015	HR: 0.696	< 0.001						
20	Moussa, 2017 [43]	Egypt	DLBCL	Serum	Healthy control	30	20	miRNA‐21	Up	24mo low: 93.33% High: 53.33%		24mo low: 26.67% high: 73.33%							
21	Nordmo, 2021 [44]	Germany	DLBCL	Tissue	—	209		miR‐130a‐3p	Down^a^	Coefficient: −0.23367		Coefficient: −0.15388							
								miR‐423‐5p	Down^a^	Coefficient: −0.06989		Coefficient: −0.13821							
								miR‐374b‐5p	Up^a^	Coefficient: 0.003091									
								miR‐590‐5p	Up^a^	Coefficient: 0.040875		Coefficient: 0.172135							
								miR‐186‐5p	Up^a^	Coefficient: 0.069885									
								miR‐106b‐5p	Up^a^	Coefficient: 0.189706		Coefficient: 0.116165							
								miR‐365a‐3p	Down^a^			Coefficient: −0.09456							
								miR‐374a‐5p	Up^a^			Coefficient: 0.098353							
22	Rinaldi, 2021 [45]	Italy	DLBCL	Serum	Healthy control	42	10	miR‐22	Down			HR: 6.89 (2.23–21.27)							
23	Roehle, 2008 [46]	Germany	DLBCL	Tissue	Nonneoplastic LN	53	7	MIRN127	Down	RR: 4.3 (1.2–15.3)	0.023							RR: 4.9 (1.7–14.1)	0.003
								MIRN21	Down	RR: 4.5 (1.4–14)	0.01								
								MIRN27A	Down	RR: 4.6 (1.5–13.6)	0.007								
								MIRNLET7G	Up									RR: 0.2 (0.1–0.6)	0.002
								MIRN19A	Down									RR: 4.2 (1.5–11.8)	0.005
24	Wang, 2020 [47]	China	GDLBCL	Tissue	Adjacent nontumor tissue	109	109	miR‐34a	Down	HR: 0.506 (0.285–0.957)	0.048	HR: 0.502 (0.28–0.961)	0.045						
25	Wang, 2014 [48]	China	DLBCL	Tissue	Reactive LN	104	28	miR‐23a	Up	HR: 3.776 (1.105–12.891)	0.034								
26	Wang, 2020 [49]	China	PGI‐DLBCL	Tissue	Adjacent nontumor tissue	84	84	miR‐150	Down	HR: 2.043 (1.147–3.638)	0.015	HR: 2.074 (1.154–3.73)	0.015						
27	Wu, 2014 [50]	China	DLBCL	Tissue	Reactive LN	106	30	miR‐146b‐5p	Down	HR: 1.267 (0.756–2.123)	0.37	HR: 1.851 (0.301–2.456)	0.046						
								miR‐320d	Down	HR: 2.002 (1.186–3.382)	0.009	HR: 2.225 (1.316–3.762)	0.003						
28	Wu, 2018 [51]	China	DLBCL	Tissue	—	82		miR‐155	Up	HR: 0.445 (0.174–1.136)	0.09	HR: 0.415 (0.193–0.891)	0.024						
29	Yang, 2018 [52]	Korea	DLBCL	Tissue	—	43		miR‐197	Down			HR: 27.9 (1.4–569)	0.031						
30	Yuan, 2016 [17]	China	DLBCL	Tissue	Healthy control	56	20	miR‐125b	Up	HR: 1.491 (1.073–2.072)	0.017								
								miR‐130a	Up	HR: 1.230 (0.938–1.624)	0.135								
31	Zheng, 2020 [53]	China	DLBCL	Tissue	—	11		miR‐155	Up	5‐year correlation: 1.742 ± 0.473									
32	Zheng, 2019 [54]	China	DLBCL	Serum	Healthy control	60	25	miR155	Up			HR: 7.035 (1.497–33.053)	0.013						
						140	75	miR155	Up			HR: 2.230 (1.183–4.202)	0.013						
33	Zhong, 2012 [55]	China	DLBCL	Tissue	Reactive LN	90	31	miR‐155	Up			HR: 0.260 (0.085–0.801)	0.019						
								miR‐146a	Up			HR: 1.119 (0.977–1.282)	0.104						

Diagnosis section													
ID	Author, year	Country	DLBCL type	Specimen	Control type	Case number	Control number	miRNA	Up/downregulation	Sensitivity	Specificity	AUC (95% CI)	*p*
1	Abou Elnour, 2022 [24]	Egypt	DLBCL	Blood	Healthy control	50	50	miR‐150	Down	0.92	0.9	0.988 (0.972–1)	< 0.001
								miR‐21	Up	0.98	0.9	0.963 (0.929–0.997)	< 0.001
2	Beheshti, 2019 [56]	USA	DLBCL	Serum	Healthy control	52	17	miR‐27a	Up	0.62	1		
								miR‐24	Up	0.85	1		
								miR‐18a	Up	0.73	1		
								miR‐15a	Up	0.75	0.94		
								miR‐155	Up	0.44	0.88		
								miR‐130a	Up	0.77	0.94		
								miR‐10b	Up	0.63	0.65		
								let‐7c	Up	0.75	1		
								let‐7b	Up	0.79	1		
3	Cao, 2022 [30]	China	DLBCL	Blood	Healthy control	123	89	miR‐379‐5p	Up	0.92	0.47	0.77 (0.7–0.82)	
								miR‐135a‐3p	Up	0.92	0.52	0.75 (0.69–0.81)	
								miR‐4476	Up	0.6	0.67	0.65 (0.59–0.72)	
								miR‐483‐3p	Down	0.81	0.54	0.71 (0.65–0.77)	
								miR‐451a	Down	0.82	0.47	0.65 (0.58–0.71)	
4	Chen, 2020 [31]	China	PGI‐DLBCL	Tissue	Reactive LN	80	46	miR‐130a	Up	0.6	0.78	0.874 (0.816–0.932)	0.03
5	Fang, 2012 [57]	China	DLBCL	Serum	Healthy control	75	77	miR‐15a	Up	0.8	0.76	0.7722 (0.6839–0.8605)	
								miR‐16‐1	Up	0.94	0.51	0.7002 (0.6027–0.7978)	
								miR‐29c	Up			0.6672 (0.5612–0.7733)	
								miR‐34a	Down	1	0.7	0.8538 (0.7714–0.9362)	
								miR‐155	Up	0.83	0.65	0.7157 (0.609–0.8224)	
6	Jorgensen, 2020 [58]	Denmark	DLBCL	Blood	Healthy control	16	14	miR‐326	Up			0.9401	
								miR‐199a‐5p	Up			0.9067	
								miR‐375	Down			0.8975	
								miR155‐5p	No difference			0.5968	
								miR215p	No difference			0.5979	
7	Khare, 2017 [6]	Israel	DLBCL	Plasma	Healthy control	14	20	miR‐124	Up			0.825	0.016
								miR‐223	Up			0.8	0.021
								miR‐532‐5p	Up			0.782	0.033
								miR‐197	Down			0.107	0.002
								miR‐345	Down			0.096	0.002
								miR‐145	Down			0.089	0.002
8	Liu, 2021 [59]	China	DLBCL	Plasma	Healthy control	42	31	miR‐107	Down			0.854	
								miR‐375‐3p	Down			0.769	
								miR‐485‐3p	Up			0.739	
9	Wang, 2014 [48]	China	DLBCL	Tissue	Reactive LN	104	28	miR‐23a	Up	0.625	0.679	0.698 (0.59–0.807)	
10	Wang, 2020 [49]	China	PGI‐DLBCL	Tissue	Adjacent nontumor tissue	84	84	miR‐150	Down	0.798	0.771	0.882	0.026
11	Xiao, 2019 [60]	China	DLBCL	Serum	Healthy control	89	48	miR‐451a	Down			0.7147 (0.6654–0.8531)	< 0.05
12	Zajdel, 2019 [61]	Poland		CNS DLBCL	Tissue	30	23	miR‐21	Up			0.714	
								miR‐19b	Up			0.737	
								miR‐92a	Up			0.771	
13	Zhong, 2012 [55]	China	DLBCL	Tissue	Reactive LN	90	31	miR‐155	Up	0.8	0.585	0.771	
								miR‐146a	Up	0.88	0.569		

Abbreviations: AUC: area under the curve, CI: confidence interval, DLBCL: diffuse large B‐cell lymphoma, HR: hazard ratio, LN: lymph node, miRNA: microRNA, OR: odds ratio, RR: relative ratio.

^a^ The expression level of these microRNAs determined by their impact on patient outcomes.

In the 44 diagnostic evaluations performed in the studies analyzed, 37 distinct microRNAs were identified. Thirty diagnostic evaluations reported microRNA upregulation, while 12 reported microRNA downregulation. The sensitivity and specificity metrics for diagnosing DLBCL have been reported by 25 diagnostic evaluations. Twenty of the 25 evaluations were conducted on distinct microRNA varieties.

In the prognostic meta‐analysis conducted, 21 distinct microRNA were evaluated. Upregulation of miRNA was observed in 46 prognostic evaluations, while downregulation was observed in 27 miRNA types.

### 3.2 Quality Assessment

The quality of the studies included in the analysis was evaluated by independent investigators using the Newcastle–Ottawa Scale (Table 2). An additional investigator was assigned the responsibility of resolving disparities in quality assessment. 45.2% of the studies received a “good” overall grade, while 54.8% received a “fair” overall score. In general, the quality assessment procedures revealed a small risk of bias in the included studies.

**TABLE 2:** The Newcastle–Ottawa Scale quality assessment.

ID	Author, year	Selection				Comparability	Exposure			Quality
		Q1	Q2	Q3	Q4	Q1	Q1	Q2	Q3	
1	Abou Elnour, 2022 [24]	*	*		*	*	*	*	*	7
2	Ahmadvand, 2018 [25]	*			*		*	*	*	5
3	Alencar, 2011 [26]	*	*			*	*			4
4	Bedewy, 2017 [27]	*	*		*	**	*	*	*	8
5	Bento, 2022 [28]	*	*			**	*			5
6	Berglund, 2012 [29]	*			*	**	*	*	*	7
7	Cao, 2022 [30]	*			*	**	*	*	*	7
8	Chen, 2020 [31]	*	*	*		**	*	*	*	8
9	Chen, 2021 [32]	*	*			**	*	*	*	7
10	Go, 2015 [33]	*	*			**	*	*	*	7
11	He, 2014 [34]	*	*		*	**	*	*	*	8
12	Hedström, 2013 [35]	*	*		*	**	*	*	*	8
13	Hu, 2021 [36]	*	*			**	*	*	*	7
14	Huang, 2022 [37]	*	*			**	*			5
15	Ji, 2022 [38]	*	*		*	**	*	*	*	8
16	Lawrie, 2007 [40]	*			*	**	*	*	*	7
17	Li, 2015 [41]	*	*		*	**	*	*	*	8
18	Marchesi, 2018 [42]	*	*		*	**	*	*		8
19	Moussa, 2017 [43]	*	*		*	**	*	*	*	8
20	Nordmo, 2021 [44]					**	*			3
21	Rinaldi, 2021 [45]	*	*		*	**	*	*	*	8
22	Roehle, 2008 [46]	*	*		*	**	*	*	*	8
23	Wang, 2020 [47]	*	*			**	*	*	*	7
24	Wang, 2014 [48]	*	*			**	*	*	*	7
25	Wang, 2020 [49]	*	*		*	**	*	*	*	8
26	Wu, 2014 [50]	*	*			**	*	*	*	7
27	Wu, 2018 [51]	*	*			**	*			5
28	Yang, 2018 [52]	*	*			**	*			5
29	Yuan, 2016 [17]	*	*		*	**	*	*	*	8
30	Zheng, 2020 [53]	*				**	*			4
31	Zheng, 2019 [54]	*	*		*	**	*	*	*	8
32	Zhong, 2012 [55]	*	*			**	*	*	*	7

### 3.3 Meta‐Analysis of the Diagnostic Value of microRNAs in DLBCL Patients

**FIGURE 2:**
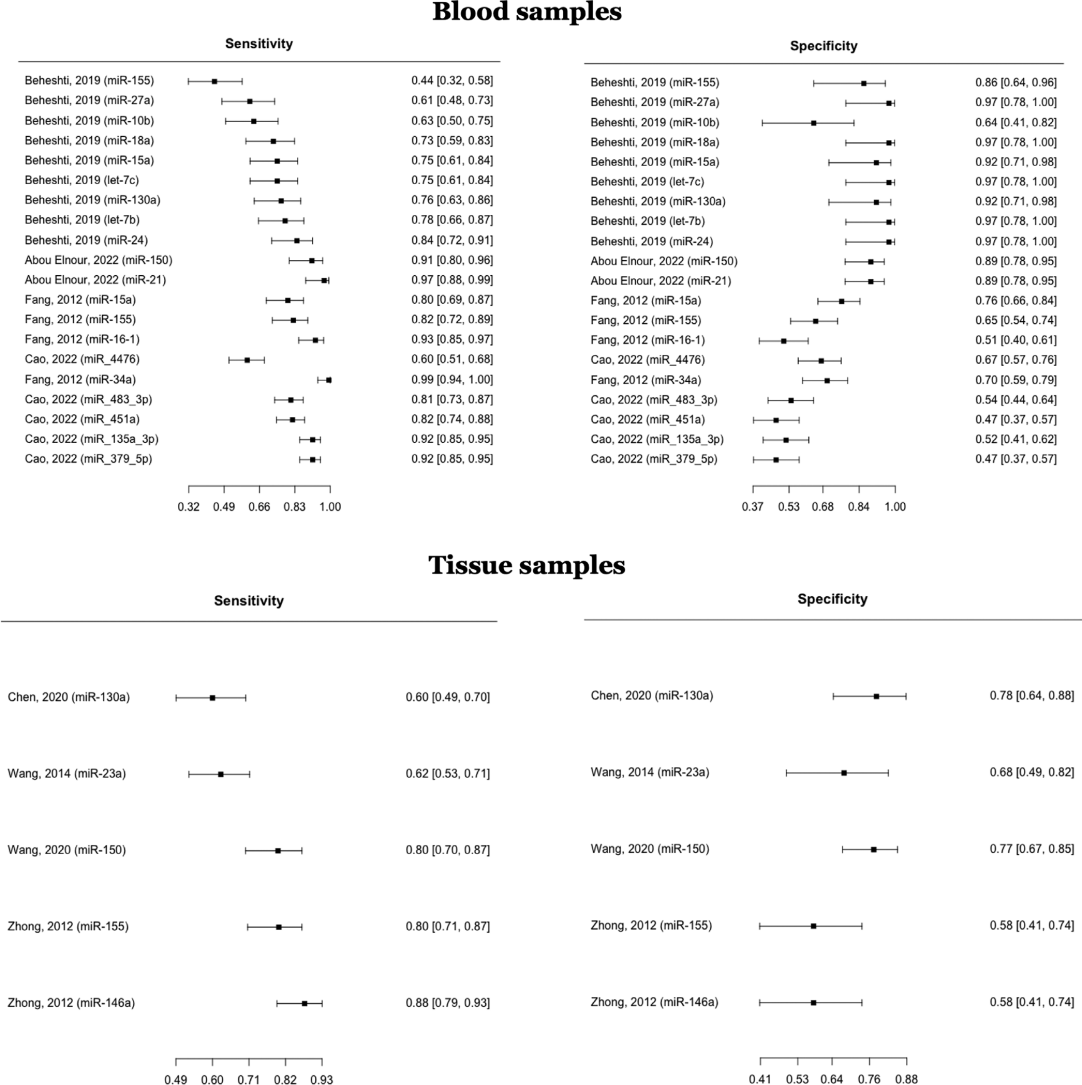
Diagnostic accuracy of microRNAs in DLBCL, bivariate random effect model meta‐analysis.

**FIGURE 3:**
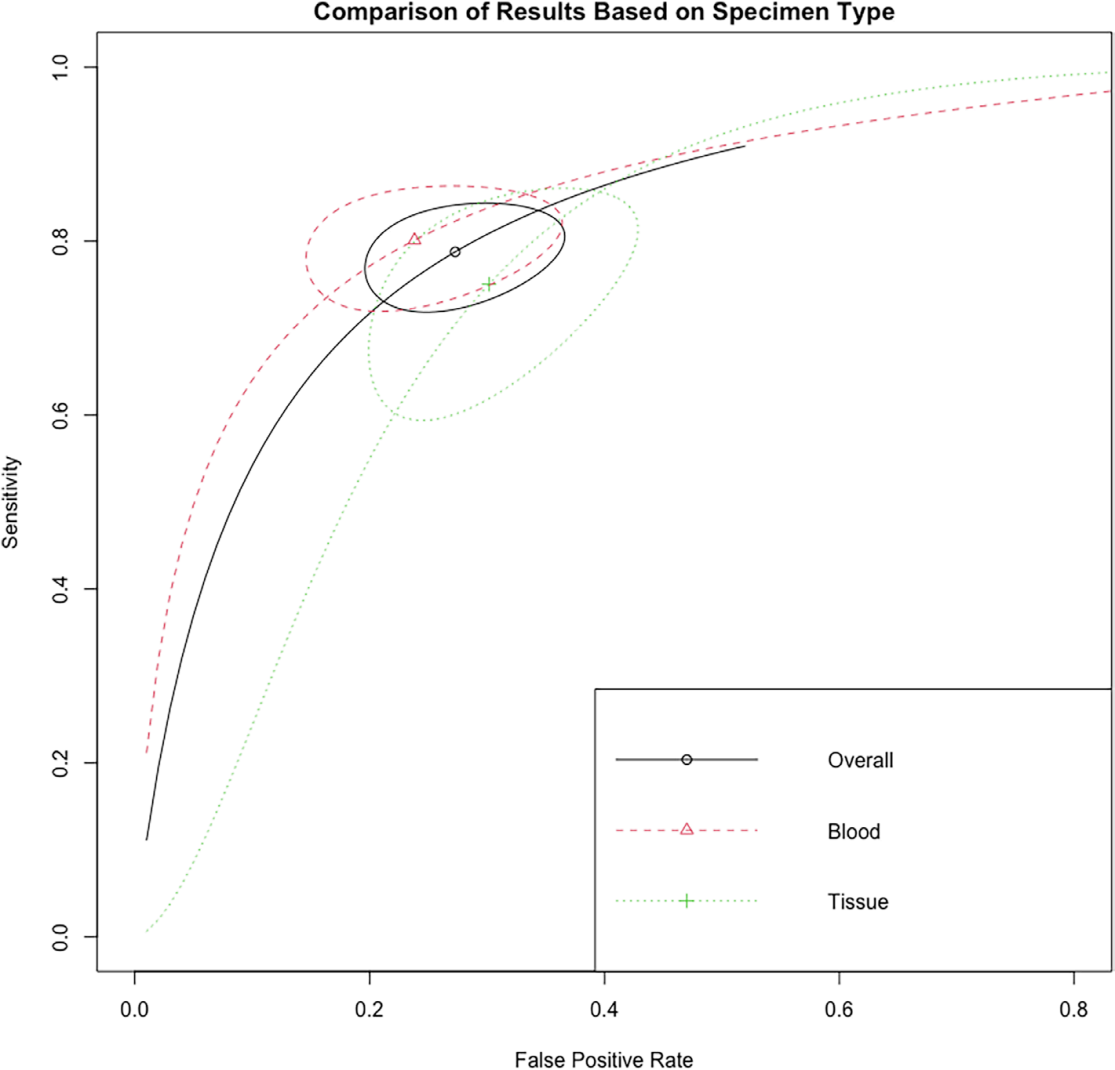
ROC curve for the diagnostic accuracy of miRNA in DLBCL.

The Reitsma bivariate model estimated a pooled sensitivity of 0.788 (95% CI: 0.733–0.834, *p* < 0.001) and a pooled specificity of 0.727 (95% CI: 0.654–0.790, *p* < 0.001) with *I* ^2^ of 61.1%–76.1% and AUC 0.824 in all types of specimens (*n* = 25), involving 1932 DLBCL cases and 1226 healthy controls. For microRNAs in blood specimens (*n* = 20), the Reitsma bivariate model indicated a cumulative sensitivity of 0.801 (95% CI: 0.737–0.853, *p* < 0.001) and a pooled specificity of 0.762 (95% CI: 0.663–0.838, *p* < 0.001) in detecting DLBCL (Figure 2). Holling sample size unadjusted *I* ^2^ was 62.9%–78.3%. The *p* value for the test for equality of sensitivities across studies was < 2e−16, while the *p* value for the test for equality of specificities was < 2e−16. After generating the SROC curve, the pooled AUC for blood specimen type was found to be 0.849 (Figure 3). MicroRNAs in tissue specimens (*n* = 5) had a cumulative sensitivity of 0.750 (95% CI: 0.628–0.843, *p* < 0.001) and a pooled specificity of 0.698 (0.598–0.783, *p* < 0.001) in diagnosing DLBCL. Holling sample size unadjusted *I* ^2^ was 55%–57.1%. The test for equality of sensitivities among the studies had a *p* value of 1.79e−05, and the test for equality of specificities had a *p* value of 0.0917. The pooled AUC for tissue specimen type was 0.773.

**FIGURE 4:**
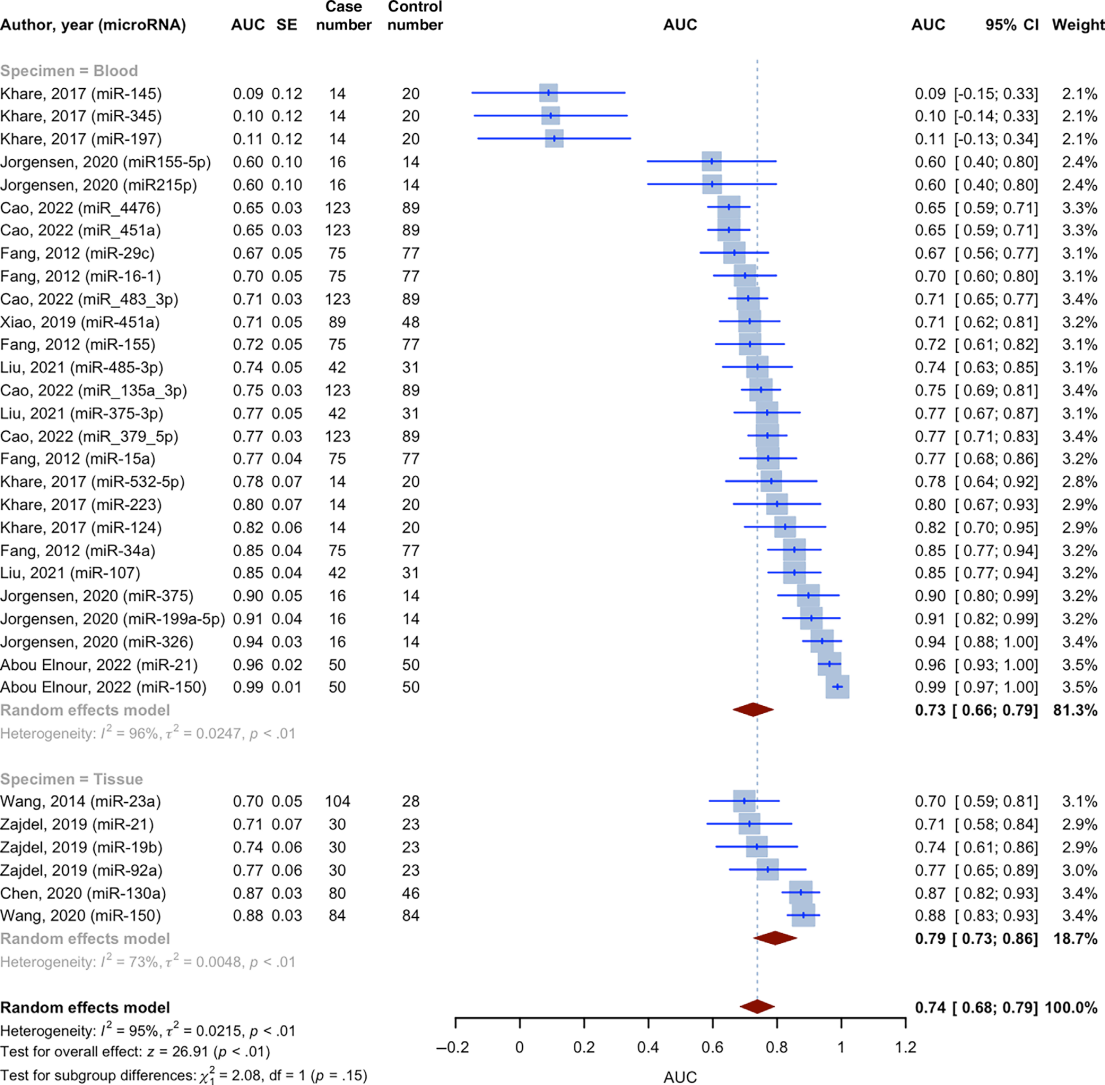
Forest plot of pooled AUCs for the diagnostic accuracy of microRNAs in DLBCL, random effect model with the inverse variance method.

The AUC values for microRNA on the identification of DLBCL were given in 33 of 44 included evaluations in the meta‐analysis involving 1827 DLBCL cases and 1488 healthy controls. Using the inverse variance approach, the pooled AUC for all was 0.7385 (95% CI: 0.6847–0.7923, *p* < 0.0001, *I* ^2^ = 95%). This value was calculated using 33 diagnostic accuracy assessments and 31 unique microRNA (Figure 4). The studies were divided into two categories depending on the type of specimen used to evaluate microRNA expression. The AUC for the blood specimen subgroup (*n* = 27) was 0.7256 (95% CI: 0.6622–0.7891; *I* ^2^ = 95.7%). The pooled AUC for the tissue specimen subgroup (*n* = 6) was 0.7942 (95% CI: 0.7259–0.8625; *I* ^2^ = 72.6%). The test for differences between subgroups was not statistically significant (*p* = 0.1495).

### 3.4 Meta‐Analysis of the Prognostic Value of microRNAs in DLBCL Patients

**FIGURE 5:**
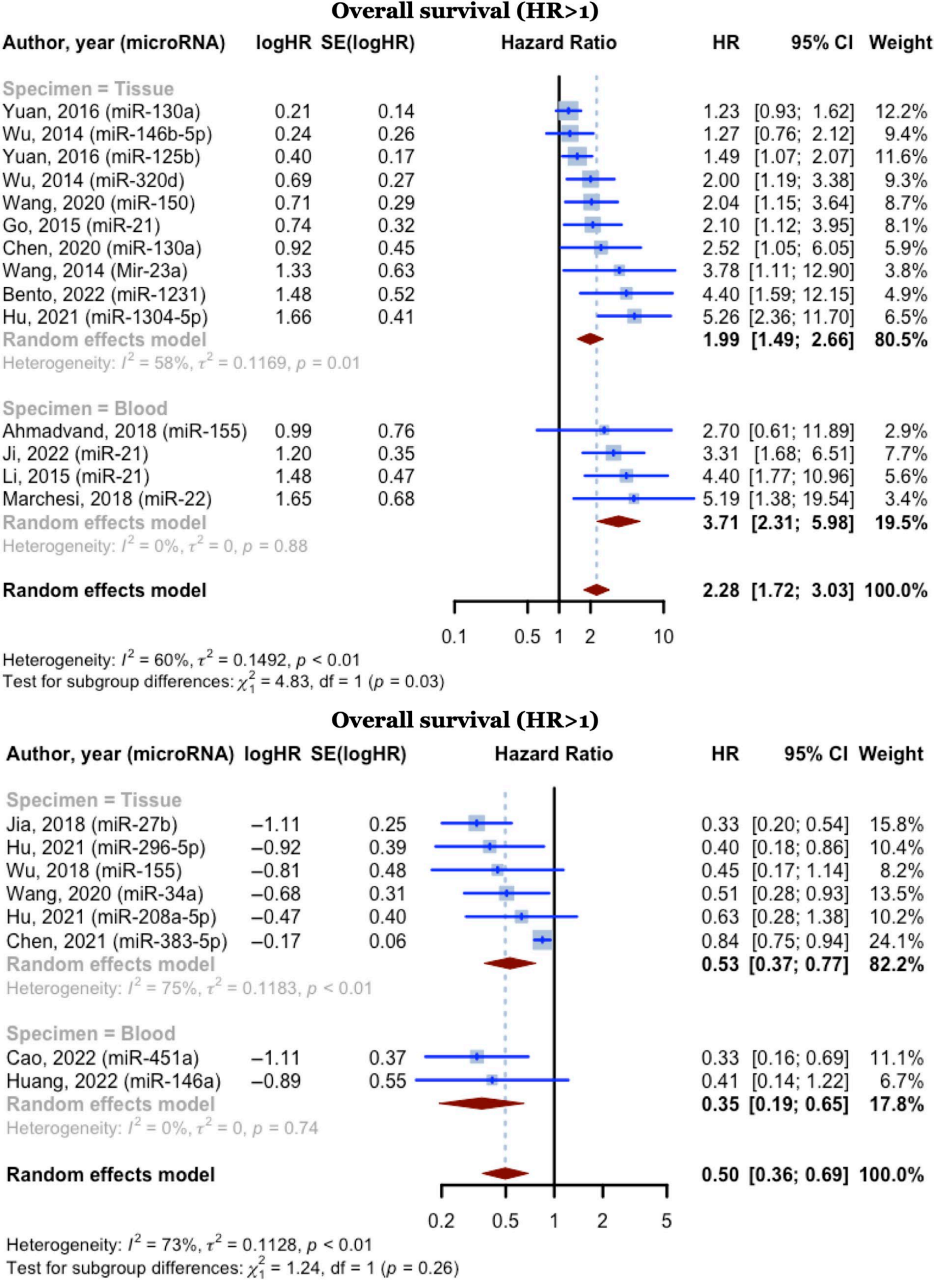
Forest plot of the overall survival hazard ratios, random effect model with the inverse variance method.

Among the 22 prognostic analyses that provided OS HRs, 14 had HR ratios higher than one and involved 1327 DLBCL cases. For these studies, the overall HR was 2.2847 (95% CI: 1.7248–3.0263, *p* < 0.0001; *I* ^2^ = 59.9%, *p =* 0.0021). The pooled HR for the blood specimen subgroup (*n* = 4) was 3.7135 (95% CI: 2.3071–5.9771; *I* ^2^ = 0.0%). The HR for the tissue specimen subgroup (*n* = 10) was 1.9879 (95% CI: 1.4869–2.6576; *I* ^2^ = 58.5%). The test for differences across subgroups was statistically significant (*p* = 0.0280). Eight studies provided HR less than one, involving 858 DLBCL cases, yielding a pooled HR of 0.4965 (95% CI: 0.3576–0.6894, *p <* 0.0001). For the blood specimen subgroup (*n* = 2), the pooled HR was 0.3545 (95% CI: 0.1930–0.6512). The HR for the subgroup of tissue specimens (*n* = 6) was 0.5310 (95% CI: 0.3678–0.7668; *I* ^2^ = 74.9%). The test for subgroup differences was not statistically significant (*p* = 0.2649) (Figure 5).

**FIGURE 6:**
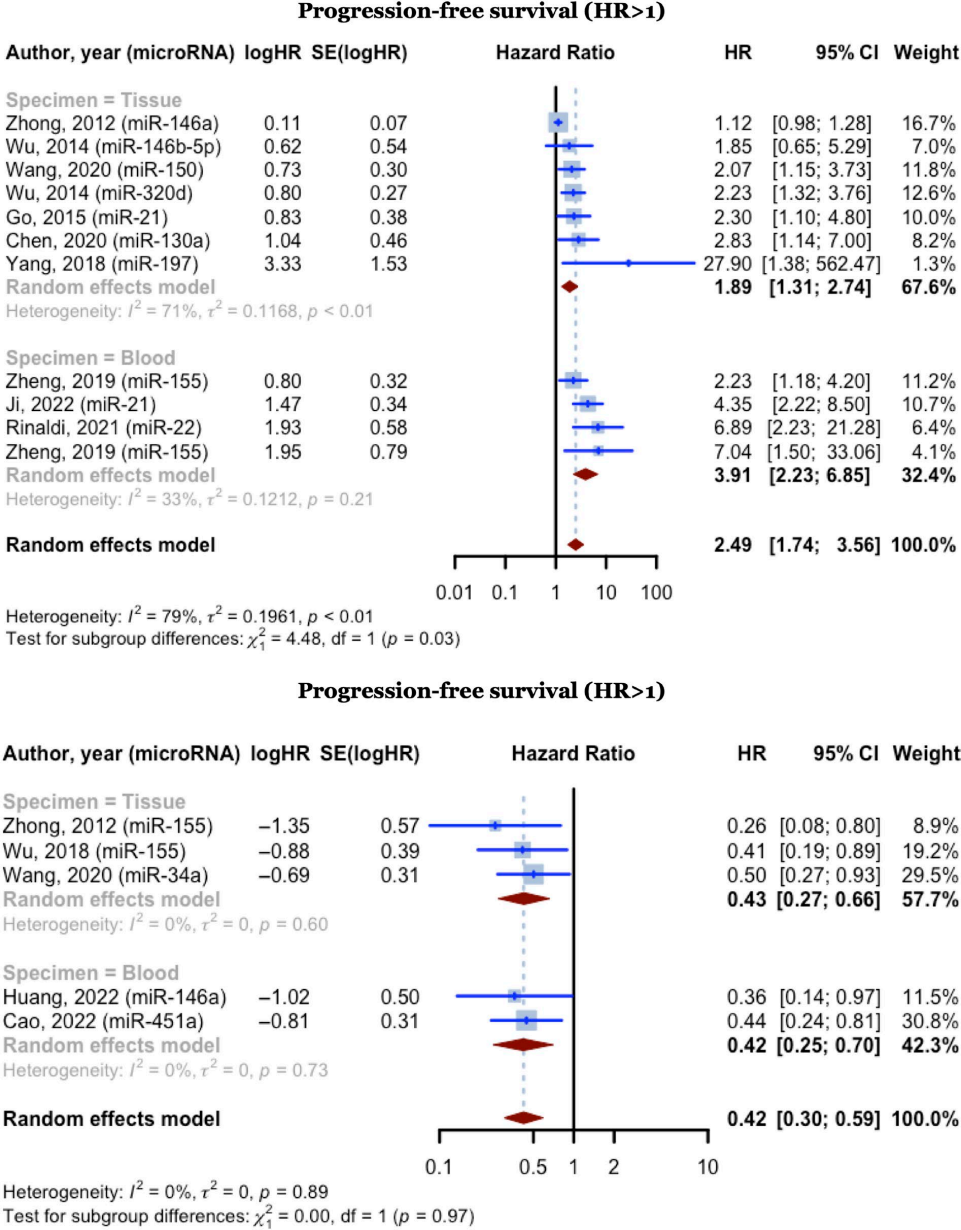
Forest plot of the progression‐free survival hazard ratios, random effect model with the inverse variance method.

Eleven of 16 PFS reporting studies exhibited HRs higher than one and included 1107 DLBCL cases. These studies had a pooled HR of 2.4883 (95% CI: 1.7367–3.5650, *p* < 0.0001; *I* ^2^ = 79.2%). In the blood specimen subgroup (*n* = 4), pooled HR was 3.9089 (95% CI: 2.2293–6.8540; *I* ^2^ = 33.2%). In the tissue specimen subgroup (*n* = 7), HR was 1.8917 (95% CI: 1.3075–2.7368; *I* ^2^ = 70.6%). The test for differences across subgroups was statistically significant (*p* = 0.0343). Five studies exhibited HRs of less than one and included 476 DLBCL cases. These studies had a pooled HR of 0.4232 (95% CI: 0.3027–0.5916, *p* < 0.0001; *I* ^2^ = 0.0%). The blood specimen subgroup (*n* = 2) had a pooled HR of 0.4199 (95% CI: 0.2509–0.7028; *I* ^2^ = 0.0%). In the tissue specimen subgroup (*n* = 3), HR was 0.4255 (95% CI: 0.2737–0.6616; *I* ^2^ = 0.0%). Subgroup difference was not significant (*p* = 0.9695) (Figure 6).

### 3.5 Diagnostic and Prognostic Features of microRNA‐155 in DLBC Patients

MiR‐155 diagnostic values have been examined in three studies. Pooling these studies involving 217 DLBCL cases and 125 controls, yielded a cumulative sensitivity of 0.710 (95% CI: 0.445–0.882, *p* > 0.1) and a pooled specificity of 0.725 (95% CI: 0.502–0.873, *p* < 0.05), with a ROC calculated AUC of 0.776. Holling *I* ^2^ for this analysis was 15.7%–15.9%. All three studies reported an upregulation in the expression of the miR‐155 in DLBCL cases compared with controls.

**FIGURE 7:**
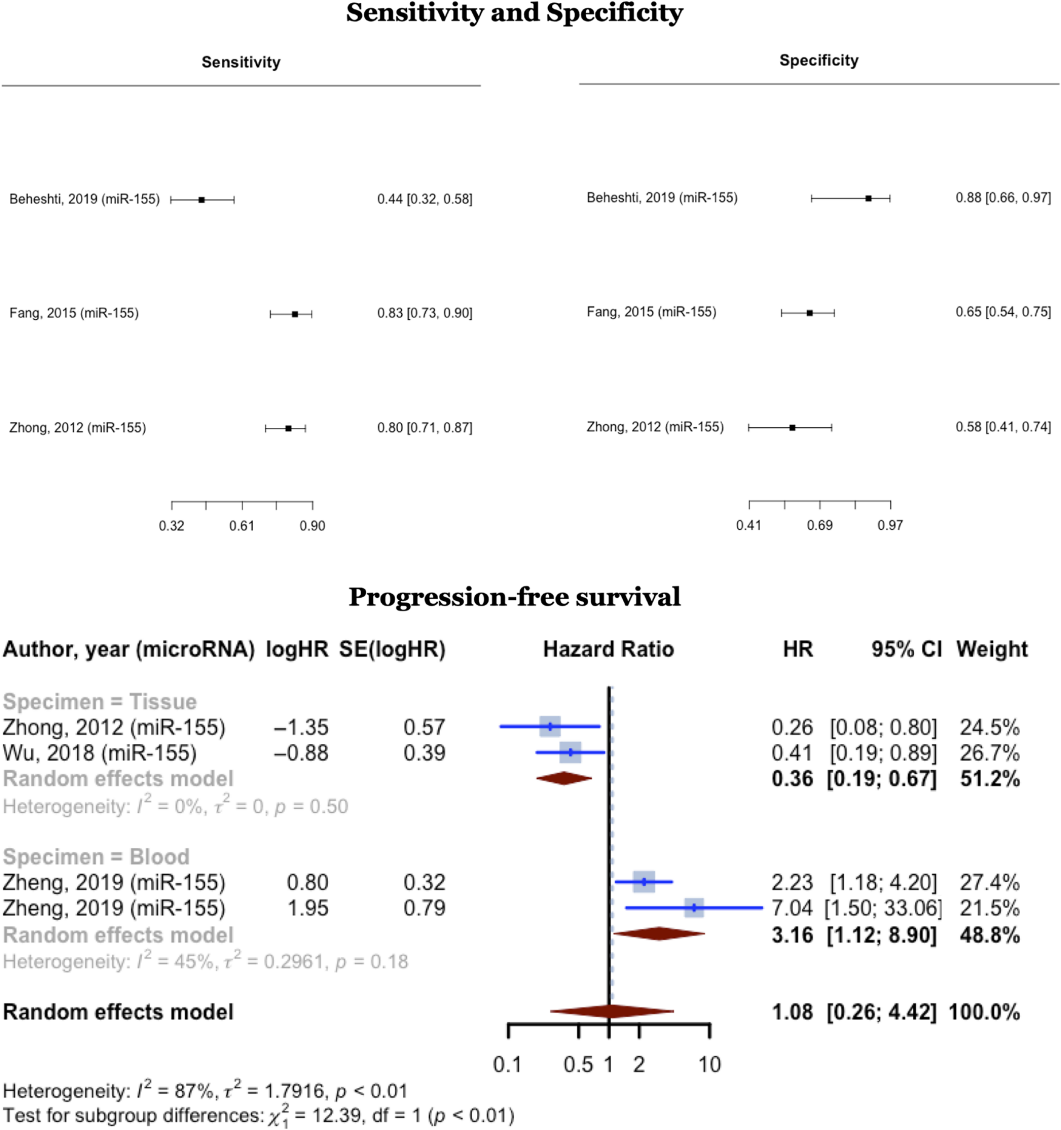
MiR‐155 sensitivity and specificity in diagnosing DLBCL and progression‐free survival hazard ratios meta‐analyses.

Four studies examined the predictive value of miR‐155 in terms of PFS. All four studies reported an upregulation in the expression of the miR‐155 in DLBCL cases. Two of these investigations used tissue samples as specimens in 172 DLBCL cases and had lower than one HR. The pooled HR for these two was 0.3578 (95% CI: 0.1902–0.6730; *I* ^2^ = 0%). The other two studies used blood samples as their specimen in 200 DLBCL cases and reported a pooled HR of 3.1604 (95% CI: 1.1223–8.8998; *I* ^2^ = 44.9%) (Figure 7).

Two other studies investigated the relationship between miR‐155 and OS. The first used tissue samples and had an HR of 0.445 (95% CI: 1.136–0.174), while the second used blood samples and had an HR of 2.7 (95% CI: 16.3–0.84).

### 3.6 Diagnostic and Prognostic Features of microRNA‐21 in DLBC Patients

Three studies examined the relationship between miR‐21 and OS. All three studies observed an HR of higher than one. One of these studies used tissue samples as their specimen, while the other two used blood samples. The three studies involved 468 DLBCL patients and reported an upregulation in miR‐21 expression. The pooled HR for these studies was 2.8938 (95% CI: 1.9157–4.3713; *I* ^2^ = 0%). Two of these studies also reported PFS for 356 DLBCL cases, with a pooled HR of 3.2217 (95% CI: 1.7291–6.0025).

**FIGURE 8:**
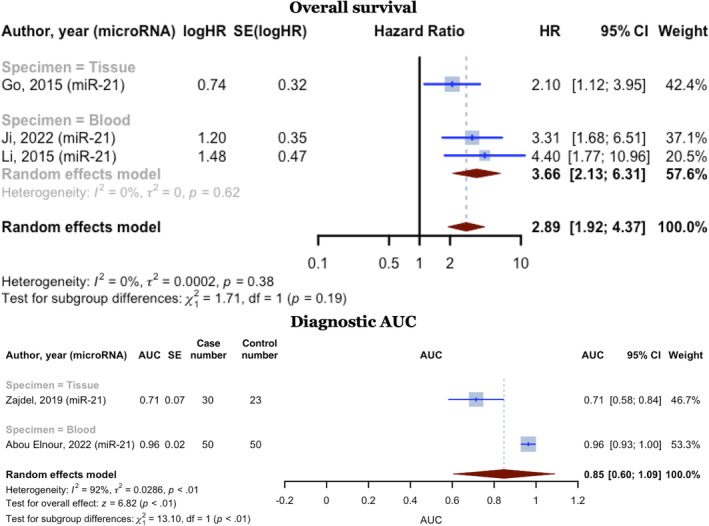
MiR‐21 overall survival hazard ratios and AUCs for diagnosing DLBCL meta‐analyses.

Two studies addressed miR‐21 diagnostic values. These studies included 80 DLBCL cases and 73 healthy controls, with one utilizing tissue samples and the other utilizing blood samples. The combined AUC for these two studies was 0.8468 (95% CI: 0.6034–1.0903, *p* < 0.0001, *I* ^2^ = 92.4%) (Figure 8).

## 4 Discussion

The high prevalence of DLBCL and challenges in its management highlight the need for novel diagnostic, prognostic, and therapeutic approaches. Our study aimed to summarize the results of current studies focusing on the diagnostic and/or prognostic value of miRNAs in patients with DLBCL (Table 3). In our included studies, the pooled AUC for all miRNAs was 0.7435 (95% CI: 0.6951–0.7918). Further subgroup analysis revealed a pooled AUC of 0.7341 (95% CI: 0.6786–0.7896) and 0.7942 (95% CI: 0.7259–0.8625) in blood and tissue specimens, respectively. Regarding the prognostic value of miRNAs in DLBCL patients, overall HR in studies with higher than one HR was 2.2847 (95% CI: 1.7248–3.0263) and 2.4883 (95% CI: 1.7367–3.5650) for OS and PFS, respectively; at the same time, in studies reporting less than one HRs, pooled HR was 0.4965 (95% CI: 0.3576–0.6894) and 0.4232 (95% CI: 0.3027–0.5916) for OS and PFS, respectively. Interestingly, in the prognostic section, a significant difference was observed in subgroup analysis based on the type of specimen (blood or tissue). This could be due to sample size, differences in the method of collecting samples, or the nature of the samples.

**TABLE 3:** Summary of results and interpretations.

Diagnosis										Prognosis					
Meta‐analysis	Specimen	Number of evaluations	Sensitivity	*p*	Specificity	*p*	AUC	*p*	*I* ^2^	Meta‐analysis	Specimen	Number of evaluations	HR	*I* ^2^	*p*
All microRNAs															
Bivariate	Blood	20	0.801 [0.737; 0.853]	< 0.001	0.762 [0.663; 0.838]	< 0.001	0.849		62.9%–78.3%	OS (HR > 1)	Blood	4	3.7135 [2.3071; 5.9771]	0.00%	
	Tissue	5	0.750 [0.628; 0.843]	< 0.001	0.698 [0.598; 0.783]	< 0.001	0.773		55%–57.1%		Tissue	10	1.9879 [1.4869; 2.6576]	58.50%	
	Total	25	0.788 [0.733; 0.834]	< 0.001	0.727 [0.654; 0.790]	< 0.001	0.824		61.1%–76.1%		Total	14	2.2847 [1.7248; 3.0263]	59.90%	< 0.0001
AUC	Blood	27					0.7256 [0.6622; 0.7891]		95.70%	OS (HR < 1)	Blood	2	0.3545 [0.1930; 0.6512]	0.00%	
	Tissue	6					0.7942 [0.7259; 0.8625]		72.60%		Tissue	6	0.5310 [0.3678; 0.7668]	74.90%	
	Total	33					0.7385 [0.6847; 0.7923]	< 0.0001	95.00%		Total	8	0.4965 [0.3576; 0.6894]	73.30%	< 0.0001
										PFS (HR > 1)	Blood	4	3.9089 [2.2293; 6.8540]	33.20%	
											Tissue	7	1.8917 [1.3075; 2.7368]	70.60%	
											Total	11	2.4883 [1.7367; 3.5650]	79.20%	< 0.0001
										PFS (HR < 1)	Blood	2	0.4199 [0.2509; 0.7028]	0.00%	
											Tissue	3	0.4255 [0.2737; 0.6616]	0.00%	
											Total	5	0.4232 [0.3027; 0.5916]	0.00%	< 0.0001
MicroRNA‐155															
Bivariate	Blood	2	0.660 [0.249; 0.919]	> 0.1	0.784 [0.465; 0.938]	> 0.05	0.794		57.8%–58.3%	PFS (HR > 1)	Blood	2	3.1604 [1.1223; 8.8998]	44.90%	
	Total	3	0.710 [0.445; 0.882]	> 0.1	0.725 [0.502; 0.873]	< 0.05	0.776		15.7%–15.9%	PFS (HR < 1)	Tissue	2	0.3578 [0.1902; 0.6730]	0.00%	
MicroRNA‐21															
AUC	Total	2					0.8468 [0.6034; 1.0903]	92.40%	< 0.0001	OS (HR > 1)	Blood	2	3.6639 [2.1285; 6.3069]	0.00%	
											Tissue	1	2.1000 [1.1153; 3.9542]	—	
											Total	3	2.8938 [1.9157; 4.3713]	0.00%	< 0.0001

Noncoding RNAs (ncRNA) are small RNA molecules which do not code proteins. MiRNAs are regulatory ncRNAs that interact with messenger RNA (mRNA) targets [62]. These molecules are unique contents of exosomes derived from cancer cells and have important roles in lymphomagenesis [63]. MiRNAs impact lymphoma progression by controlling cell growth, proliferation, differentiation, survival, and apoptosis [63]. The detectability of tumor‐associated miRNAs in body fluids such as serum or plasma [64] and their stability which may be due to EVs [65], make miRNAs a potential noninvasive prognostic, diagnostic, and therapeutic biomarkers in various diseases.

A study by Lawrie et al., which was the first study that investigated miRNA in DLBCL, showed that plasma levels of miR‐21, miR‐155, and miR‐210 were upregulated in DLBCL patients and expression of miR‐21 was associated with RFS [66]. Emerging evidence has reported miRNAs as biomarkers in DLBCL. Several studies that did not meet our inclusion criteria reported the diagnostic role of various miRNAs; for instance, a study on 20 DLBCL patients showed that miR‐3960, miR‐6089, and miR‐939‐5p could be used as the diagnostic miRNA biomarkers in DLBCL [67]. In a study by Meng, Quan, Liu, 51 differently expressed miRNAs were identified in serum samples from DLBCL patients [16]. Another study revealed that miR‐4284 and miR‐4484 have the potential to be diagnostic biomarkers to help differentiate between DLBCL and reactive lymph node hyperplasia [68].

In addition to their diagnostic role, miRNAs can be used as prognostic biomarkers in DLBCL. For example, a higher expression level of miR‐155 was observed in aggressive forms of DLBCL, and it was associated with shorter EFS [69, 70]. A study by Shepshelovich et al. identified eight miRNAs that were associated with poor prognosis in DLBCL patients; of these miRNAs, the strongest correlation was observed in miR‐342‐3p and miR‐150‐5p [71]. Additionally, Montes‐Moreno et al. created a predictive model for DLBCL patients by using a microarray to identify differentially‐expressed miRNAs in which nine miRNAs were evaluated and a predictive model based on miRNAs expression was applied for predicting OS and PFS [72].

MiR‐155 diagnostic values have been evaluated in four studies. Pooling three studies resulted in a cumulative sensitivity of 0.710 and a pooled specificity of 0.725 with an ROC‐calculated AUC of 0.776. Interestingly, a higher expression of miR‐155 in tissue samples was associated with better survival (PFS), while increased levels of miR‐155 in blood samples were associated with shorter PFS. DLBCL cells have higher copy numbers of miR‐155 than normal circulating B cells [73]. A study by Zhang et al. showed that the FOXP3 level in DLBCL cell lines decreased after silencing miR‐155. Thus, miR‐155 may function through the regulation of FOXP3 [74]. Also, it has been revealed that miR‐155 increases the proliferation of B‐cell lymphoma cells and inhibits apoptosis by inhibiting FOCXO3 [53]. Complete recovery occurred in mice models when the miR‐155 stimulus was reversed [74]. In the study of Wu et al., overexpression of miR‐155 in DLBCL was associated with shorter PFS [51]. A higher level of this miRNA was detected in aggressive DLBCL forms, correlating with unfavorable clinical characteristics [69, 70].

MiR‐21 is recognized as an oncogenic miRNA activated by Nuclear Factor Kappa B (NF‐κB), which in turn downregulates various phosphatases including programmed cell death protein 4 (PDCD4) [75] and phosphatase and tensin homolog (PTEN) [76], which play crucial roles in signaling pathways such as phosphatidylinositol 3‐kinase/protein kinase B (PI3K/AKT) and mitogen‐activated protein kinase (MAPK). In a study by Chen et al., miR‐21 expression was found to be elevated in a DLBCL cell line [64]. A study on DLBCL cell lines revealed that inhibition of this miRNA increases apoptosis and induces suppression of proliferation and invasion [77, 78]. In our included studies, the pooled HR was 2.8938 (95% CI: 1.9157–4.3713) and 3.2217 (95% CI: 1.7291–6.0025) for OS and PFS, respectively. In these studies, increased miR‐21 expression in both serum and tissue has been linked to shorter survival times [33]. However, high expression of this miRNA in serum samples of DLBCL patients was associated with a better prognosis in the study conducted by Chen et al. [64].

Furthermore, the expression profile of miRNAs varies in different types of lymphoma, which could be beneficial, especially when differentiating between DLBCL and Burkitt lymphoma (BL) is required [79]. Differences in miRNA profiles among various types of lymphoma may arise from distinct miRNA expressions inherent to the cell of origin or specific defects associated with each lymphoproliferative disorder [80]. In one study, 19 miRNAs have shown different expressions in DLBCL and BL [79]. In another study, miRNA signature in BL significantly differed from DLBCL, while only minor differences were observed in miRNA profiling among three variants of BL, including sporadic BL (sBL), endemic BL (eBL), and human immunodeficiency virus‐associated BL (HIV‐BL) [81]. These findings support the possible usefulness of miRNA profiling in diagnosing DLBCL. Incorporating these miRNAs into routine practice may enhance patient stratification, allowing for more personalized treatment plans. For instance, miRNA profiles could help predict responses to specific therapies, monitor treatment efficacy, and detect disease recurrence earlier. However, several challenges must be addressed for successful implementation, including the need for standardized testing protocols, the variability of miRNA expression across different patient cohorts, and the necessity for further validation in larger, multicenter studies. Additionally, integrating miRNA testing into clinical workflows will require training for healthcare professionals to accurately interpret and apply miRNA data in decision‐making.

To the best of our knowledge, our study represents the first systematic review and meta‐analysis examining the prognostic and diagnostic value of miRNAs in DLBCL. However, several limitations should be acknowledged. First, the analysis was constrained by the limited number of miRNAs examined across multiple studies, which restricts the generalizability of our findings to specific miRNA types. Second, significant methodological differences among the included studies such as variations in sample types, outcome measures, detection methods, and follow‐up durations contributed to heterogeneity in the results, potentially affecting the consistency and reliability of our conclusions. Additionally, while RT‐qPCR is a standard method for miRNA detection, variations in cut‐off values across studies may have influenced result variability, leading to inconsistent interpretations. Furthermore, our review exclusively focused on studies that utilized samples directly from human subjects, thereby excluding relevant data derived from databases, which may limit the comprehensiveness of our analysis. Last, due to data insufficiency we could not consider important clinical factors like patient demographics, comorbidities, and treatment regimens that may influence miRNA expression and its prognostic implications.

## 5 Conclusion

A comprehensive understanding of various miRNAs and their expression profiles holds significant potential as a robust diagnostic and prognostic tool in DLBCL. Emerging evidence strongly supports the inclusion of miRNAs as novel biomarkers for enhancing the management and outcomes of DLBCL patients in the future. Future studies with larger sample sizes should focus on clinical application of miRNAs and their diagnostic and prognostic roles, particularly miR‐155 and miR‐21, in monitoring treatment responses in DLBCL, aiming to enhance personalized therapy and improve patient outcomes.

## Author Contributions

**Shaghayegh Khanmohammadi:** conceptualization, methodology, investigation, writing – original draft, writing – review and editing, project administration, supervision. **Mahdi Masrour:** methodology, conceptualization, investigation, writing – original draft, writing – review and editing. **Parisa Fallahtafti:** methodology, writing – original draft, writing – review and editing. **Fatemeh Hasani:** writing – review and editing.

## Disclosure

This article was independently researched and written, with no conflicts of interest or funding sources that could impact its content. Any opinions expressed in the article are solely those of the authors.

## Ethics Statement

The authors have nothing to report.

## Consent

The authors have nothing to report.

## Conflicts of Interest

The authors declare no conflicts of interest.

## Supporting information


**Table S1.** Excluded studies after full text review.

**Data S1.** Search strategy.

Data S2.

## Data Availability

The data that supports the findings of this study are available in the Supporting Information of this article.
