# Supplementary material for: MicroRNA as a Potential Diagnostic and Prognostic Biomarker in Diffuse Large B‐Cell Lymphoma: A Systematic Review and Meta‐Analysis
Source: Cancer Rep (Hoboken). 2025 Jan 24;8(1):e70070. doi: 10.1002/cnr2.70070 (PMC11760998; doi:10.1002/cnr2.70070)
Supplement: Supplementary file 1 — Table S1. Excluded studies after full text review. [file CNR2-8-e70070-s002.docx]

**Table 1.** Excluded studies after full text review.

| **ID** | **Author, year** | **Title** | **Reason of exclusion** |
| --- | --- | --- | --- |
|  | V. Caner, 2021 (1) | The miRNA content of circulating exosomes in DLBCL patients and in vitro influence of DLBCL-derived exosomes on miRNA expression of healthy B-cells from peripheral blood | No related survival/diagnostic performance analysis |
|  | H. Asker, 2021 (2) | Prognostic values of microRNA-21 and Ki-67 in diffuse large B-Cell lymphoma patients: Egyptian experience | Full text unavailable |
|  | Y. N. Guo, 2012 (3) | Significance of micro RNA-21 expression in diffuse large B-cell lymphoma | Full text unavailable |
|  | F. Asmar, 2014 (4) | Diffuse large B-cell lymphoma with combined TP53 mutation and MIR34A methylation: Another "double hit" lymphoma with very poor outcome? | Combined data with other mutations |
|  | M. Battistella, 2015 (5) | The high expression of the microRNA 17-92 cluster and its paralogs, and the downregulation of the target gene PTEN, is associated with primary cutaneous B-Cell lymphoma progression | Combined data with other miRNAs |
|  | N. M. Borges, 2016 (6) | Angiomirs expression profiling in diffuse large B-Cell lymphoma | No related survival/diagnostic performance analysis |
|  | A. Caivano, 2017 (7) | MicroRNA-155 in serum-derived extracellular vesicles as a potential biomarker for hematologic malignancies - a short report | No related survival/diagnostic performance analysis |
|  | C. Bouvy, 2018 (8) | Circulating microRNAs as biomarkers in diffuse large B-cell lymphoma: A pilot prospective longitudinal clinical study | No related survival/diagnostic performance analysis |
|  | G. Song, 2014 (9) | Deregulated expression of miR-224 and its target gene: CD59 predicts outcome of diffuse large B-cell lymphoma patients treated with R-CHOP | Full text unavailable |
|  | W. Chen, 2014 (10) | Clinical significance and detection of microRNA-21 in serum of patients with diffuse large B-cell lymphoma in Chinese population | Data was reported as charts and the numbers were not given. |
|  | L. Di Lisio, 2012 (11) | MicroRNA signatures in B-cell lymphomas | No related survival/diagnostic performance analysis |
|  | H. Due, 2020 (12) | MicroRNAs associated to single drug components of R-CHOP identifies diffuse large B-cell lymphoma patients with poor outcome and adds prognostic value to the international prognostic index | Data from dataset |
|  | H. Due, 2019 (13) | MicroRNA-155 controls vincristine sensitivity and predicts superior clinical outcome in diffuse large B-cell lymphoma | Data from dataset |
|  | Y. Feng, 2019 (14) | Exosome-derived miRNAs as predictive biomarkers for diffuse large B-cell lymphoma chemotherapy resistance | Data was reported as charts and the numbers were not given. |
|  | J. Fu, 2021 (15) | MicroRNA‑196a‑3p inhibits cell proliferation and promotes cell apoptosis by targeting ADP ribosylation factor 4 in diffuse large B‑cell lymphoma | Data was reported as charts and the numbers were not given. |
|  | Y. Gao, 2021 (16) | MiR-145-5p exerts anti-tumor effects in diffuse large B-cell lymphoma by regulating S1PR1/STAT3/AKT pathway | No related survival/diagnostic performance analysis |
|  | Y. Huang, 2019 (17) | MiR‑101 regulates the cell proliferation and apoptosis in diffuse large B‑cell lymphoma by targeting MEK1 via regulation of the ERK/MAPK signaling pathway | Data was reported as charts and the numbers were not given. |
|  | Y. Huang, 2019 (18) | MiR-101 regulates cell proliferation and apoptosis by targeting KDM1A in diffuse large B cell lymphoma | No related survival/diagnostic performance analysis |
|  | J. Iqbal, 2015 (19) | Global microRNA expression profiling uncovers molecular markers for classification and prognosis in aggressive B-cell lymphoma | Data was reported as charts and the numbers were not given. |
|  | I. Jung, 2009 (20) | MicroRNA-155 expression and outcome in diffuse large B-cell lymphoma | Correspondence study |
|  | V. Kakkassery, 2017 (21) | Vitreous microRNA levels as diagnostic biomarkers for vitreoretinal lymphoma | Letter to editor |
|  | J. Kang, 2020 (22) | Systematic analysis of competing endogenous RNA networks in diffuse large B-Cell lymphoma and Hodgkin's lymphoma | Data from dataset |
|  | S. Knudsen, 2015 (23) | Development and blind clinical validation of a microRNA based predictor of response to treatment with R-CHO(E)P in DLBCL | No related survival/diagnostic performance analysis |
|  | C. H. Lawrie, 2009 (24) | Expression of microRNAs in diffuse large B cell lymphoma is associated with immunophenotype, survival and transformation from follicular lymphoma | Data was reported as charts and the numbers were not given. |
|  | C. H. Lawrie, 2008 (25) | Detection of elevated levels of tumour-associated microRNAs in serum of patients with diffuse large B-cell lymphoma | Data was reported as charts and the numbers were not given. |
|  | E. L. Lim, 2015 (26) | Comprehensive miRNA sequence analysis reveals survival differences in diffuse large B-cell lymphoma patients | Insufficient data |
|  | J. Liu, 2020 (27) | Exosomal miR-107 as novel biomarker and tumor suppressor by targeting Ywhah in diffuse large B-Cell lymphoma | Abstract |
|  | J. Liu, 2021 (28) | Serum exosomal micrornas as novel noninvasive biomarkers for diffuse large b-cell lymphoma | Abstract |
|  | F. Marchesi, 2021 (29) | Serum mir-22 as novel non-invasive predictor of clinical outcome and response to therapy in patients with diffuse large b-cell lymphoma | Abstract |
|  | F. Marchesi, 2018 (30) | Serum MIR-22 as novel non-invasive predictor of poor clinical outcome in patients with diffuse large B-Cell lymphoma: preliminary results of an ongoing prospective study | Abstract |
|  | F. Marchesi, 2017 (31) | Liquid biopsy: Deciphering a signature of circulating micrornas as novel non-invasive biomarkers in diffuse large b-cell lymphoma | Abstract |
|  | S. C. Marques, 2016 (32) | High miR-34a expression improves response to doxorubicin in diffuse large B-cell lymphoma | Insufficient data |
|  | S. Montes-Moreno, 2012 (33) | Risk adapted-high dose therapies modulate the impact of biological classification in Diffuse Large B cell lymphoma prognosis. Analysis of biological markers in patients from clinical trials in geltamo and gotel Spanish collaborative groups | Abstract |
|  | S. Montes-Moreno, 2011 (34) | MiRNA expression in diffuse large B-cell lymphoma treated with chemoimmunotherapy | Data was reported as charts and the numbers were not given. |
|  | M. Moussa, 2017 (35) | The diagnostic and prognostic implications of circulating mirna-21 in a sample of hepatitis c/none hepatitis diffuse large b-cell lymphoma Egyptian patients | Abstract |
|  | H. D. Munch-Petersen, 2016 (36) | TP53 hotspot mutations are predictive of survival in primary central nervous system lymphoma patients treated with combination chemotherapy | No related survival/diagnostic performance analysis |
|  | H. D. Munch-Petersen, 2015 (37) | Differential expression of miR-155 and miR-21 in tumor and stroma cells in diffuse large B-cell lymphoma | Data was reported as charts and the numbers were not given. |
|  | H. Ni, 2015 (38) | Low expression of miRNA-224 predicts poor clinical outcome in diffuse large B-cell lymphoma treated with R-CHOP | Insufficient data |
|  | Z. Y. Niu, 2017 (39) | The polymorphism at the miRNA binding site of GOLGA(7) is associated with the Non-Hodgkin's lymphoma cancer risk | No related survival/diagnostic performance analysis |
|  | M. Rahouma, 2019 (40) | Prognostic value of microRNA-21/ Ki-67 in non-Hodgkin's lymphoma: NCI experience | Abstract |
|  | S. Rui, 2019 (41) | Serum microrna prognostic model and underlying immune alterations in diffuse large B cell lymphoma | Abstract |
|  | A. Sharma, 2021 (42) | Mir-671-5p, mir-193b-5p, mir-1307-5p are useful for predicting outcome in diffuse large B-cell lymphoma | Abstract |
|  | D. Shepshelovich, 2015 (43) | MicroRNA signature is indicative of long term prognosis in diffuse large B-cell lymphoma | Insufficient data |
|  | Y. Shi, 2019 (44) | Reproducibility of quantitative real-time PCR analysis in microRNA expression profiling and comparisons with microarray assays in diffuse large B-cell lymphoma patients | Abstract |
|  | C. Y. Ting, 2022 (45) | Downregulation of hsa-miR-548d-3p and overexpression of HOXA9 in diffuse large B-cell lymphoma patients and the risk of R-CHOP chemotherapy resistance and disease progression | No related survival/diagnostic performance analysis |
|  | T. Stopka, 2011 (46) | Pattern of Mir-155 and PU.1 expression in CLL/SLL and aggressive lymphomas | Abstract |
|  | S. Suthandiram, 2015 (47) | Differential expression of micrornas in the serum of patients with diffuse large b-cell lymphoma | Abstract |
|  | G. Tamaddon, 2016 (48) | Mir-4284 and mir-4484 as putative biomarkers for diffuse large B-cell lymphoma | No data |
|  | W. Tang, 2019 (49) | Pre-miR-27a rs895819 polymorphism and risk of diffuse large B-cell lymphoma | No related survival/diagnostic performance analysis |
|  | N. Tau, 2010 (50) | Characterizing microrna signature as a prognostic factor in diffuse large b cell lymphoma | Abstract |
|  | D. R. Thapa, 2014 (51) | Serum microRNAs in HIV-infected individuals as pre-diagnosis biomarkers for AIDS-NHL | No related survival/diagnostic performance analysis |
|  | G. Song, 2014 (52) | Serum microRNA expression profiling predict response to R-CHOP treatment in diffuse large B cell lymphoma patients | No related survival/diagnostic performance analysis |
|  | K. Troppan, 2014 (53) | High expression of miRNA-199A-1 and miRNA-497-1 is associated with better overall survival in aggressive non-Hodgkin's lymphoma | Abstract |
|  | K. Troppan, 2015 (54) | MiR-199a and miR-497 are associated with better overall survival due to increased chemosensitivity in aggressive non-Hodgkin's lymphoma patients | Abstract |
|  | E. Voropaeva, 2023 (55) | Tumor-specific methylation of p53-responsive oncosuppressive microRNA genes in Diffuse Large B-cell Lymphoma | Abstract |
|  | E. Voropaeva, 2022 (56) | Methylation of p53-responsive oncosuppressive microrna genes in diffuse large Bcell lymphoma tumor tissue | Abstract |
|  | E. Voropaeva, 2021 (57) | Tumor-nonspecific methylation of the mir- 145 gene in diffuse large B-cell lymphoma | Abstract |
|  | E. N. Voropaeva, 2022 (58) | The methylation of the p53 targets the genes mir-203, mir-129-2, mir-34a and mir-34b/c in the tumor tissue of diffuse large B-cell lymphoma | No related survival/diagnostic performance analysis |
|  | J. Wang, 2013 (59) | Significance of microrna-146b-5p in diffuse large B-cell lymphoma and its relation to risk assessment | Abstract |
|  | J. Wang, 2012 (60) | Significance of microRNA-223 in DLBCL and its relation to prognosis | Abstract |
|  | X. Wang, H, 2013 (61) | Microrna-17∼92 cluster upregulates NF-KB activity via suppressing multiple NF-KB negative regulators mediating ubiquitination | Abstract |
|  | X. Wu, 2017 (62) | Novel bio-markers latent membrane protein 1 and microrna-155 for the prognostic prediction of diffuse large b cell lymphoma | Abstract |
|  | C. Xu, 2018 (63) | Circulating exsomal MIR-451A for therapy response monitoring in diffuse large B cell lymphoma | Abstract |
|  | M. Xu, 2018 (64) | Expression and clinical significance of miR-23a and MTSS1 in diffuse large B-cell lymphoma | Insufficient data |
|  | B. Yang, C, 2014 (65) | A polymorphism at the microRNA binding site in the 3 ' untranslated region of C14orf101 is associated with non-Hodgkin lymphoma overall survival | No related survival/diagnostic performance analysis |
|  | S. Yoshizawa, 2010 (66) | Circulating MIR-92A level is a novel biomarker for monitoring patients with non-Hodgkin's lymphoma | Abstract |
|  | N. Zare, 2019 (67) | The expression level of hsa-miR-146a-5p in plasma-derived exosomes of patients with diffuse large B-cell lymphoma | Insufficient data |
|  | N. Zare, 2019 (68) | Evaluation of exosomal miR-155, let-7g and let-7i levels as a potential noninvasive biomarker among refractory/relapsed patients, responsive patients and patients receiving R-CHOP | Insufficient data |
|  | Z. Zheng, 2017 (69) | MiR21 sensitized B-lymphoma cells to ABT-199 via ICOS/ICOSL-mediated interaction of Treg cells with endothelial cells | Insufficient data |
|  | H. Zhong, 2011 (70) | Clinical significance and prognosis of Mir-155 and Mir-146a expression levels in formalin-fixed/paraffin-embedded tissue of patients with diffuse large B-cell lymphoma | Abstract |
|  | H. Zhuang, 2014 (71) | MicroRNA-146a rs2910164 polymorphism and the risk of diffuse large B cell lymphoma in the Chinese Han population | No related survival/diagnostic performance analysis |
|  | M. Alhanafy, 2020 (72) | Clinical implications of serum mi-RNA -155 and mi-RNA- 92a in diffuse large B cell lymphoma | Abstract |
|  | J. Alencar, 2009 (73) | MicroRNA Are Useful Biomarkers for Prediction of Response to Therapy and Survival of Patients with Diffuse Large B-Cell Lymphoma | Abstract |
|  | N. J. Bahlis, 2010 (74) | A miRNA Risk Score for the Prediction of Response to Rituximab-CHOP Therapy and Survival of Patients with Diffuse Large B-Cell Lymphoma | Abstract |
|  | A. Beheshti, 2018 (75) | Ultra-Sensitive Detection of Circulating Serum microRNAs (miRNAs) in Diffuse Large B-Cell Lymphoma (DLBCL) Patient-Derived Xenograft (PDX) Models and Correlation with Disease Status in DLBCL Patient | Abstract |
|  | L. Bento, 2019 (76) | Analysis of Micro-RNAs Associated to Treatment Failure in Diffuse Large B Cell Lymphoma | Abstract |
|  | N. M. Borges, 2012 (77) | Pro-angiogenic mir-296 is frequently overexpressed and is associated with advanced stage disease in diffuse large B-cell lymphoma | Abstract |
|  | R. E. Culpin, 2013 (78) | Micrornas of the miR-17-92 cluster predict for outcome in RCHOP-treated diffuse large B-cell lymphoma and are significantly associated with expression of MYC protein | Abstract |
|  | C. Di, 2018 (79) | Circulating Exosomal microRNA Signature As a Noninvasive Biomarker for Diagnosis of Diffuse Large B-Cell Lymphoma | Abstract |
|  | C. Hother, 2012 (80) | MiRNA profiling predicts survival and identifies a novel putative oncomir in diffuse large B-cell lymphoma treated with immunochemotherapy | Abstract |
|  | R. Culpin, 2011 (81) | Mature microRNAs of the miR-17-92 cluster predict for disease outcome in diffuse large B-cell lymphoma (DLBCL) patients treated with CHOP-R immunochemotherapy | Abstract |
|  | H. Due, 2016 (82) | Low expression of MIR-155 in vincristine resistant diffuse large B-cell lymphoma | Abstract |
|  | K. Dybkær, 2019 (83) | Addition of Drug-Response Specific Micro-RNAs to the International Prognostic Index Improves Prognostic Stratification of GCB-DLBCL Patients Treated with R-CHOP | Abstract |
|  | Fassina, 2013 (84) | The diagnostic value of miR17-92 microRNA cluster in diffuse large B-cell lymphoma | Abstract |
|  | H. Go, 2014 (85) | MiR-21 has strong prognostic implications and functions as an oncogenic miR by modulating PI3K/Akt pathway at multiple levels in diffuse large B cell lymphoma | Abstract |
|  | S. F. Gohar, 2018 (86) | The impact of serum microRNA-21 on outcome of diffuse large B-cell lymphoma patients | Abstract |
|  | K. Inada, 2014 (87) | Availability of Circulating microRNAs As a Biomarker for the Early Diagnosis of Diffuse Large B-Cell Lymphoma | Abstract |
|  | S. Jørgensen, 2014 (88) | Plasma microrna predicts B-cell lymphoma up to 12 months before diagnosis-Data from the Danish blood donor study | Abstract |
|  | M. Kushnir, 2010 (89) | Prognostic value of microRNAs expression in diffuse large B cell lymphoma | Abstract |
|  | M. Lahav, 2008 (90) | MicroRNA Is Prognostic Indicator in Patients with Diffuse Large B Cell Lymphoma | Abstract |
|  | E. Paszkiewicz-Kozik, 2021 (91) | Peripheral Blood Cells from Patients with Hodgkin's and Diffuse Large B Cell Lymphomas May Be a Better Source of Candidate Diagnostic miRNAs Than Circulating miRNAs | No related survival/diagnostic performance analysis |
|  | R. Sun, 2021 (92) | A novel prognostic model based on four circulating miRNA in diffuse large B-cell lymphoma: implications for the roles of MDSC and Th17 cells in lymphoma progression | Combined data with other miRNAs |

1. Caner V, Cetin GO, Hacioglu S, Baris IC, Tepeli E, Turk NS, et al. The miRNA content of circulating exosomes in DLBCL patients and in vitro influence of DLBCL-derived exosomes on miRNA expression of healthy B-cells from peripheral blood. Cancer Biomark. 2021;32(4):519-29.

2. Asker H, Khorshed E, Ahmed M, Refaat L, Khaled H, Rashed R. Prognostic Values of MicroRNA-21 and Ki-67 in Diffuse Large B-Cell Lymphoma Patients: Egyptian Experience. CLINICAL LABORATORY. 2021;67(7):1697-705.

3. Guo YN, Wang JQ, Meng D, Guo JM, Zhong GP, Yu WY, et al. Significance of micro RNA-21 expression in diffuse large B-cell lymphoma. Journal of Leukemia and Lymphoma. 2012;21(5):269-72.

4. Asmar F, Hother C, Kulosman G, Treppendahl MB, Nielsen HM, Ralfkiaer U, et al. Diffuse large B-cell lymphoma with combined TP53 mutation and MIR34A methylation: Another "double hit" lymphoma with very poor outcome? ONCOTARGET. 2014;5(7):1912-25.

5. Battistella M, Romero M, Castro-Vega LJ, Gapihan G, Bouhidel F, Bagot M, et al. The High Expression of the microRNA 17-92 Cluster and its Paralogs, and the Downregulation of the Target Gene PTEN, Is Associated with Primary Cutaneous B-Cell Lymphoma Progression. J Invest Dermatol. 2015;135(6):1659-67.

6. Borges NM, Elias MV, Fook-Alves VL, Andrade TA, de Conti ML, Macedo MP, et al. Angiomirs expression profiling in diffuse large B-Cell lymphoma. Oncotarget. 2016;7(4):4806-16.

7. Caivano A, La Rocca F, Simeon V, Girasole M, Dinarelli S, Laurenzana I, et al. MicroRNA-155 in serum-derived extracellular vesicles as a potential biomarker for hematologic malignancies - a short report. Cell Oncol (Dordr). 2017;40(1):97-103.

8. Bouvy C, Wannez A, George F, Graux C, Chatelain C, Dogné JM. Circulating MicroRNAs as Biomarkers in Diffuse Large B-cell Lymphoma: A Pilot Prospective Longitudinal Clinical Study. Biomark Cancer. 2018;10:1179299x18781095.

9. Song G, Song G, Ni H, Gu L, Liu H, Chen B, et al. Deregulated expression of miR-224 and its target gene: CD59 predicts outcome of diffuse large B-cell lymphoma patients treated with R-CHOP. Curr Cancer Drug Targets. 2014;14(7):659-70.

10. Chen W, Wang H, Chen H, Liu S, Lu H, Kong D, et al. Clinical significance and detection of microRNA-21 in serum of patients with diffuse large B-cell lymphoma in Chinese population. Eur J Haematol. 2014;92(5):407-12.

11. Di Lisio L, Sánchez-Beato M, Gómez-López G, Rodríguez ME, Montes-Moreno S, Mollejo M, et al. MicroRNA signatures in B-cell lymphomas. Blood Cancer J. 2012;2(2):e57.

12. Due H, Brøndum RF, Young KH, Bøgsted M, Dybkær K. MicroRNAs associated to single drug components of R-CHOP identifies diffuse large B-cell lymphoma patients with poor outcome and adds prognostic value to the international prognostic index. BMC Cancer. 2020;20(1):237.

13. Due H, Schönherz AA, Ryø L, Primo MN, Jespersen DS, Thomsen EA, et al. MicroRNA-155 controls vincristine sensitivity and predicts superior clinical outcome in diffuse large B-cell lymphoma. Blood Adv. 2019;3(7):1185-96.

14. Feng Y, Zhong M, Zeng S, Wang L, Liu P, Xiao X, et al. Exosome-derived miRNAs as predictive biomarkers for diffuse large B-cell lymphoma chemotherapy resistance. Epigenomics. 2019;11(1):35-51.

15. Fu J, Lou X, Wan S, Zhao X, Chen Z, Zhu M, et al. microRNA‑196a‑3p inhibits cell proliferation and promotes cell apoptosis by targeting ADP ribosylation factor 4 in diffuse large B‑cell lymphoma. Oncol Rep. 2021;45(2):764-75.

16. Gao Y, Ding X. miR-145-5p exerts anti-tumor effects in diffuse large B-cell lymphoma by regulating S1PR1/STAT3/AKT pathway. Leuk Lymphoma. 2021;62(8):1884-91.

17. Huang Y, Zou Y, Lin L, Ma X, Zheng R. miR‑101 regulates the cell proliferation and apoptosis in diffuse large B‑cell lymphoma by targeting MEK1 via regulation of the ERK/MAPK signaling pathway. Oncol Rep. 2019;41(1):377-86.

18. Huang Y, Zou Y, Lin L, Ma X, Zheng R. MiR-101 regulates cell proliferation and apoptosis by targeting KDM1A in diffuse large B cell lymphoma. Cancer Management and Research. 2019;11:2739-46.

19. Iqbal J, Shen Y, Huang X, Liu Y, Wake L, Liu C, et al. Global microRNA expression profiling uncovers molecular markers for classification and prognosis in aggressive B-cell lymphoma. Blood. 2015;125(7):1137-45.

20. Jung I, Aguiar RC. MicroRNA-155 expression and outcome in diffuse large B-cell lymphoma. Br J Haematol. 2009;144(1):138-40.

21. Kakkassery V, Schroers R, Coupland SE, Wunderlich MI, Schargus M, Heinz C, et al. Vitreous microRNA levels as diagnostic biomarkers for vitreoretinal lymphoma. Blood. 2017;129(23):3130-3.

22. Kang J, Yao P, Tang Q, Wang Y, Zhou Y, Huang J. Systematic Analysis of Competing Endogenous RNA Networks in Diffuse Large B-Cell Lymphoma and Hodgkin's Lymphoma. Front Genet. 2020;11:586688.

23. Knudsen S, Hother C, Gronbaek K, Jensen T, Hansen A, Mazin W, et al. Development and Blind Clinical Validation of a MicroRNA Based Predictor of Response to Treatment with R-CHO(E)P in DLBCL. PLOS ONE. 2015;10(2).

24. Lawrie CH, Chi J, Taylor S, Tramonti D, Ballabio E, Palazzo S, et al. Expression of microRNAs in diffuse large B cell lymphoma is associated with immunophenotype, survival and transformation from follicular lymphoma. J Cell Mol Med. 2009;13(7):1248-60.

25. Lawrie CH, Gal S, Dunlop HM, Pushkaran B, Liggins AP, Pulford K, et al. Detection of elevated levels of tumour-associated microRNAs in serum of patients with diffuse large B-cell lymphoma. Br J Haematol. 2008;141(5):672-5.

26. Lim EL, Trinh DL, Scott DW, Chu A, Krzywinski M, Zhao Y, et al. Comprehensive miRNA sequence analysis reveals survival differences in diffuse large B-cell lymphoma patients. Genome Biol. 2015;16(1):18.

27. Liu J, Han Y, Hu S, Zhan L, Hu X, Yang J, et al. Exosomal MiR-107 As Novel Biomarker and Tumor Suppressor By Targeting Ywhah in Diffuse Large B-Cell Lymphoma. Blood. 2020;136:26-7.

28. Liu J, Zhou X, Cai Y, Hu S, Lu T, Ren S, et al. Serum exosomal micrornas as novel noninvasive biomarkers for diffuse large b-cell lymphoma. HemaSphere. 2021;5(SUPPL 2):401-2.

29. Marchesi F, Regazzo G, Palombi F, Tremante E, Bertoni F, Terrenato I, et al. Serum mir-22 as novel non-invasive predictor of clinical outcome and response to therapy in patients with diffuse large b-cell lymphoma. HemaSphere. 2021;5(SUPPL 2):409.

30. Marchesi F, Regazzo G, Sacconi A, Palombi F, Tremante E, Terrenato I, et al. Serum MIR-22 As Novel Non-Invasive Predictor of Poor Clinical Outcome in Patients with Diffuse Large B-Cell Lymphoma: Preliminary Results of An Ongoing Prospective Study. Haematologica. 2020;105(SUPPL 2):S101.

31. Marchesi F, Regazzo G, Sacconi A, Spagnuolo M, Donzelli S, Palombi F, et al. LIQUID BIOPSY: DECIPHERING A SIGNATURE OF CIRCULATING MICRORNAS AS NOVEL NON-INVASIVE BIOMARKERS IN DIFFUSE LARGE B-CELL LYMPHOMA. HAEMATOLOGICA. 2017;102:566-7.

32. Marques SC, Ranjbar B, Laursen MB, Falgreen S, Bilgrau AE, Bødker JS, et al. High miR-34a expression improves response to doxorubicin in diffuse large B-cell lymphoma. Exp Hematol. 2016;44(4):238-46.e2.

33. Montes-Moreno S, Batlle A, De Villambrosia SG, Sanchez-Espiridión B, Cereceda L, González-Barca E, et al. Risk adapted-high dose therapies modulate the impact of biological classification in Diffuse Large B cell lymphoma prognosis. Analysis of biological markers in patients from clinical trials in geltamo and gotel Spanish collaborative groups. Blood. 2012;120(21).

34. Montes-Moreno S, Martinez N, Sanchez-Espiridión B, Díaz Uriarte R, Rodriguez ME, Saez A, et al. miRNA expression in diffuse large B-cell lymphoma treated with chemoimmunotherapy. Blood. 2011;118(4):1034-40.

35. Moussa M, Elhalawani N, Nazir A, Mashali N, Nafea MH, Sorour A. THE DIAGNOSTIC AND PROGNOSTIC IMPLICATIONS OF CIRCULATING MIRNA-21 IN A SAMPLE OF HEPATITIS C/NONE HEPATITIS DIFFUSE LARGE B-CELL LYMPHOMA EGYPTIAN PATIENTS. HAEMATOLOGICA. 2017;102:691-.

36. Munch-Petersen HD, Asmar F, Dimopoulos K, Areškevičiūtė A, Brown P, Girkov MS, et al. TP53 hotspot mutations are predictive of survival in primary central nervous system lymphoma patients treated with combination chemotherapy. Acta Neuropathol Commun. 2016;4:40.

37. Munch-Petersen HD, Ralfkiaer U, Sjö LD, Hother C, Asmar F, Nielsen BS, et al. Differential expression of miR-155 and miR-21 in tumor and stroma cells in diffuse large B-cell lymphoma. Appl Immunohistochem Mol Morphol. 2015;23(3):188-95.

38. Ni H, Wang X, Liu H, Tian F, Song G. Low expression of miRNA-224 predicts poor clinical outcome in diffuse large B-cell lymphoma treated with R-CHOP. Biomarkers. 2015;20(4):253-7.

39. Niu ZY, Sun SY, Li GX. The polymorphism at the miRNA binding site of GOLGA(7) is associated with the Non-Hodgkin's lymphoma cancer risk. INTERNATIONAL JOURNAL OF CLINICAL AND EXPERIMENTAL PATHOLOGY. 2017;10(3):3735-8.

40. Rahouma M, Rashed RA, Asker HA, Abdel-Azim LR, Naguib E, Khaled H. Prognostic value of microRNA-21/ Ki-67 in non-Hodgkin's lymphoma: NCI experience. Annals of Oncology. 2019;30:v437.

41. Rui S, Zhao W. Serum microRNA Prognostic Model and Underlying Immune Alterations in Diffuse Large B Cell Lymphoma. Blood. 2019;134:5225.

42. Sharma A, Das A, Bal A, Srinivasan R, Malhotra P, Prakash G, et al. Mir-671-5p, Mir-193b-5p, Mir-1307-5p Are Useful for Predicting Outcome in Diffuse Large B-Cell Lymphoma. Blood. 2021;138:2399.

43. Shepshelovich D, Ram R, Uziel O, Kushnir M, Lithwick-Yanai G, Hoshen M, et al. MicroRNA signature is indicative of long term prognosis in diffuse large B-cell lymphoma. Leuk Res. 2015;39(6):632-7.

44. Shi Y, Liu TY, Song MY, Chen L, Liu J, Gao S. Reproducibility of quantitative real-time PCR analysis in microRNA expression profiling and comparisons with microarray assays in diffuse large B-cell lymphoma patients. INTERNATIONAL JOURNAL OF CLINICAL AND EXPERIMENTAL MEDICINE. 2019;12(5):5776-84.

45. Ting CY, Tan SY, Gan GG, Zain SM, Pung YF, Ong DB, et al. Downregulation of hsa-miR-548d-3p and overexpression of HOXA9 in diffuse large B-cell lymphoma patients and the risk of R-CHOP chemotherapy resistance and disease progression. Int J Lab Hematol. 2022;44(5):907-17.

46. Stopka T, Vargova K, Huskova H, Burda P, Curik N, Vlckova P, et al. Pattern of Mir-155 and PU.1 expression in CLL/SLL and aggressive lymphomas. Blood. 2011;118(21).

47. Suthandiram S, Gan GG, Zain SM, Bee PC, Lian LH, Chang KM, et al. Differential Expression of microRNAs in the Serum of Patients with Diffuse Large B-Cell Lymphoma. PUBLIC HEALTH GENOMICS. 2015;18:28-.

48. Tamaddon G, Geramizadeh B, Karimi MH, Mowla SJ, Abroun S. miR-4284 and miR-4484 as Putative Biomarkers for Diffuse Large B-Cell Lymphoma. Iran J Med Sci. 2016;41(4):334-9.

49. Tang W, Xu H, Ma D, Ma R, Wu J, Yu X, et al. Pre-miR-27a rs895819 polymorphism and risk of diffuse large B-cell lymphoma. J Clin Lab Anal. 2020;34(3):e23088.

50. Tau N, Lahav M. Characterizing microrna signature as a prognostic factor in diffuse large b cell lymphoma. Israel Medical Association Journal. 2010;12(10):646.

51. Thapa DR, Hussain SK, Tran WC, D'Souza G, Bream JH, Achenback CJ, et al. Serum MicroRNAs in HIV-infected individuals as pre-diagnosis biomarkers for AIDS-NHL. Journal of Acquired Immune Deficiency Syndromes. 2014;66(2):229-37.

52. Song G, Gu L, Li J, Tang Z, Liu H, Chen B, et al. Serum microRNA expression profiling predict response to R-CHOP treatment in diffuse large B cell lymphoma patients. Ann Hematol. 2014;93(10):1735-43.

53. Troppan K, Wenzl K, Deutsch A, Pichler M, Graupp M, Beham-Schmid C, et al. High expression of miRNA-199A-1 and miRNA-497-1 is associated with better overall survival in aggressive nonhodgkin's lymphoma. Haematologica. 2014;99:135.

54. Troppan K, Wenzl K, Pichler M, Pursche B, Schwarzenbacher D, Feichtinger J, et al. MiR-199a and miR-497 are associated with better overall survival due to increased chemosensitivity in aggressive non-Hodgkin's lymphoma patients. Oncology Research and Treatment. 2015;38:63-4.

55. Voropaeva E, Churkina M, Pospelova T, Gurazheva A, Maksimov V, Berezina O. Tumor-specific methylation of p53-responsive oncosuppressive microRNA genes in Diffuse Large B-cell Lymphoma. European Journal of Human Genetics. 2023;31:161.

56. Voropaeva E, Pospelova T, Churkina M, Berezina O, Gurazheva A, Maximov V. METHYLATION OF P53-RESPONSIVE ONCOSUPPRESSIVE MICRORNA GENES IN DIFFUSE LARGE BCELL LYMPHOMA TUMOR TISSUE. HemaSphere. 2022;6:3752.

57. Voropaeva E, Pospelova T, Churkina M, Maximov V, Gurageva A. Tumor-nonspecific methylation of the mir- 145 gene in diffuse large b-cell lymphoma. HemaSphere. 2021;5(SUPPL 2):764.

58. Voropaeva EN, Pospelova TI, Orlov YL, Churkina MI, Berezina OV, Gurazheva AA, et al. The Methylation of the p53 Targets the Genes MIR-203, MIR-129-2, MIR-34A and MIR-34B/C in the Tumor Tissue of Diffuse Large B-Cell Lymphoma. Genes (Basel). 2022;13(8).

59. Wang J. Significance of microrna-146b-5p in diffuse large B-cell lymphoma and its relation to risk assessment. Hematological Oncology. 2013;31:210.

60. Wang J, Su L, Xi Y, Yao X. Significance of microRNA-223 in DLBCL and its relation to prognosis. Histopathology. 2012;61:127.

61. Wang X, Wang H, Bi C, Zhang X, Huang X, Zhang X, et al. Microrna-17∼92 cluster upregulates NF-KB activity via suppressing multiple NF-KB negative regulators mediating ubiquitination. Blood. 2015;126(23):3638.

62. Wu X, Wang F, Li Y, Zhang H, Liu P, Wang X, et al. Novel bio-markers latent membrane protein 1 and microrna-155 for the prognostic prediction of diffuse large b cell lymphoma. Blood. 2017;130.

63. Xu C, Cao D, Jiang Y, Xu J, Feng Y, Jing C. Circulating exsomal MIR-451A for therapy response monitoring in diffuse large B cell lymphoma. HemaSphere. 2018;2:813-4.

64. Xu M, Xu TAO. Expression and clinical significance of miR-23a and MTSS1 in diffuse large B-cell lymphoma. Oncology Letters. 2018;16(1):371-7.

65. Yang B, Liu C, Diao LP, Wang CJ, Guo ZJ. A polymorphism at the microRNA binding site in the 3 ' untranslated region of C14orf101 is associated with non-Hodgkin lymphoma overall survival. CANCER GENETICS. 2014;207(4):141-6.

66. Yoshizawa S, Ohyashiki K, Umezu T, Kurada M, Ohyashiki J. Circulating MIR-92A level is a novel biomarker for monitoring patients with non-Hodgkin's lymphoma. Haematologica. 2010;95:171.

67. Zare N, Eskandari N, Mehrzad V, Javanmard S. The expression level of hsa-miR-146a-5p in plasma-derived exosomes of patients with diffuse large B-cell lymphoma. Journal of Research in Medical Sciences. 2019;24(1).

68. Zare N, Haghjooy Javanmard S, Mehrzad V, Eskandari N, Kefayat A. Evaluation of exosomal miR-155, let-7g and let-7i levels as a potential noninvasive biomarker among refractory/relapsed patients, responsive patients and patients receiving R-CHOP. Leuk Lymphoma. 2019;60(8):1877-89.

69. Zheng Z, Xu PP, Wang L, Zhao HJ, Weng XQ, Zhong HJ, et al. MiR21 sensitized B-lymphoma cells to ABT-199 via ICOS/ICOSL-mediated interaction of Treg cells with endothelial cells. J Exp Clin Cancer Res. 2017;36(1):82.

70. Zhong H. Clinical significance and prognosis of Mir-155 and Mir-146a expression levels in formalin-fixed/paraffin-embedded tissue of patients with diffuse large B-cell lymphoma. Blood. 2011;118(21).

71. Zhuang H, Shen J, Zheng Z, Luo X, Gao R, Zhuang X. MicroRNA-146a rs2910164 polymorphism and the risk of diffuse large B cell lymphoma in the Chinese Han population. Med Oncol. 2014;31(12):306.

72. Alhanafy AM, Abou-Elnour E, El-Naidany S, Mohammed O. Clinical implications of serum mi-RNA -155 and mi-RNA- 92a in diffuse large B cell lymphoma. Annals of Oncology. 2020;31:S298.

73. Alencar AJ, Malumbres R, Advani R, Talreja N, Shyam R, Briones J, et al. MicroRNA Are Useful Biomarkers for Prediction of Response to Therapy and Survival of Patients with Diffuse Large B-Cell Lymphoma. BLOOD. 2009;114(22):258-9.

74. Bahlis NJ, Owen CJ, Neri P, Mansoor A, Gratton KJ, Duggan P, et al. A miRNA Risk Score for the Prediction of Response to Rituximab-CHOP Therapy and Survival of Patients with Diffuse Large B-Cell Lymphoma. BLOOD. 2010;116(21):147-8.

75. Beheshti A, Stevenson KE, Dashnamoorthy R, Vanderburg C, Weinstock DM, Evens AM. Ultra-Sensitive Detection of Circulating Serum microRNAs (miRNAs) in Diffuse Large B-Cell Lymphoma (DLBCL) Patient-Derived Xenograft (PDX) Models and Correlation with Disease Status in DLBCL Patient. BLOOD. 2018;132.

76. Bento L, Ros T, Muncunill J, Asensio V, Fernández C, Quintero AM, et al. Analysis of Micro-RNAs Associated to Treatment Failure in Diffuse Large B Cell Lymphoma. Blood. 2019;134:1627.

77. Borges NM, De Conti ML, De Andrade TA, Macedo MP, De Souza Begnami MDF, Alves AC, et al. Pro-angiogenic mir-296 is frequently overexpressed and is associated with advanced stage disease in diffuse large B-cell lymphoma. Blood. 2012;120(21).

78. Culpin RE, Sieniawski M, Anderson JJ, Angus B, Proctor SJ, Kometa ST, et al. Micrornasof the miR-17-92 cluster predict foroutcomein RCHOP-treated diffuse large B-cell lymphoma and are significantly associated with expression of MYC protein. Hematological Oncology. 2013;31:157.

79. Di C, Jiang Y, Li M, Juan X, Xu CG. Circulating Exosomal microRNA Signature As a Noninvasive Biomarker for Diagnosis of Diffuse Large B-Cell Lymphoma. BLOOD. 2018;132.

80. Hother C, Reker D, Dimopoulos K, Knudsen S, Jensen T, Møller MB, et al. MiRNA profiling predicts survival and identifies a novel putative oncomir in diffuse large B-cell lymphoma treated with immunochemotherapy. Blood. 2012;120(21).

81. Culpin R, Sieniawski M, Anderson J, Angus B, Proctor S, Menon G, et al. Mature microRNAs of the miR-17-92 cluster predict for disease outcome in diffuse large B-cell lymphoma (DLBCL) patients treated with CHOP-R immunochemotherapy. INTERNATIONAL JOURNAL OF MOLECULAR MEDICINE. 2011;28:S12-S.

82. Due H, Amanda Schönherz A, Bechmann Krogh L, Støve Bødker J, Schmitz A, Froberg Brøndum R, et al. Low expression of MIR-155 in vincristine resistant diffuse large B-cell lymphoma. Haematologica. 2016;101:398.

83. Dybkær K, Due H, Brøndum RF, Young KH, Bøgsted M. Addition of Drug-Response Specific Micro-RNAs to the International Prognostic Index Improves Prognostic Stratification of GCB-DLBCL Patients Treated with R-CHOP. Blood. 2019;134:1623.

84. Fassina A, Cappellesso R, Marino F, Siri M, Simonato F, Benetti M, et al. The diagnostic value of miR17-92 microRNA cluster in diffuse large B-cell lymphoma. VIRCHOWS ARCHIV. 2013;463(2):135-6.

85. Go H, Jang JY, Nam SJ, Kim YG, Paik JH, Kim TM, et al. MiR-21 has strong prognostic implications and functions as an oncogenic miR by modulating PI3K/Akt pathway at multiple levels in diffuse large B cell lymphoma. Cancer Research. 2014;74(19).

86. Gohar SF, Kamal Eldin S, El-Bassal F, Shehata A, Azzam A, Tawfik E, et al. The impact of serum microRNA-21 on outcome of diffuse large B-cell lymphoma patients. Annals of Oncology. 2018;29:viii367.

87. Inada K, Okoshi Y, Cho Y, Saito H, Iijima T, Hori M, et al. Availability of Circulating microRNAs As a Biomarker for the Early Diagnosis of Diffuse Large B-Cell Lymphoma. BLOOD. 2014;124(21).

88. Jørgensen S, Tholstrup D, Hansen JW, Keld HM, Hjalgrim H, De Nully Brown P, et al. Plasma microrna predicts B-cell lymphoma up to 12 months before diagnosis-Data from the Danish blood donor study. Blood. 2014;124(21).

89. Kushnir M, Lahav M, Ram R, Gilad S, Zepeniuk M, Cohen L, et al. Prognostic value of microRNAs expression in diffuse large B cell lymphoma. Cancer Research. 2010;70(8).

90. Lahav M, Ram R, Kushnir M, Spilberg O, Yanai GL, Beeri O, et al. MicroRNA Is Prognostic Indicator in Patients with Diffuse Large B Cell Lymphoma. BLOOD. 2008;112(11):299-300.

91. Paszkiewicz-Kozik E, Paziewska A, Kulecka M, Dabrowska M, Kluska A, Balabas A, et al. Peripheral Blood Cells from Patients with Hodgkin's and Diffuse Large B Cell Lymphomas May Be a Better Source of Candidate Diagnostic miRNAs Than Circulating miRNAs. BIOMED RESEARCH INTERNATIONAL. 2021;2021.

92. Sun R, Zheng Z, Wang L, Cheng S, Shi Q, Qu B, et al. A novel prognostic model based on four circulating miRNA in diffuse large B-cell lymphoma: implications for the roles of MDSC and Th17 cells in lymphoma progression. Mol Oncol. 2021;15(1):246-61.
