# Supplementary material for: MicroRNA as a Potential Diagnostic and Prognostic Biomarker in Diffuse Large B‐Cell Lymphoma: A Systematic Review and Meta‐Analysis
Source: Cancer Rep (Hoboken). 2025 Jan 24;8(1):e70070. doi: 10.1002/cnr2.70070 (PMC11760998; doi:10.1002/cnr2.70070)
Supplement: Supplementary file 2 — Data S1. Search strategy. [file CNR2-8-e70070-s003.docx]

**Search strategy:**

**PubMed:**

(Lymphoma, Large B-Cell, Diffuse[MeSH] OR “Lymphoma, Large B-Cell, Diffuse”[tiab] OR “Lymphoma, Histiocytic”[tiab] OR “Histiocytic Lymphomas”[tiab] OR “Lymphoma, Large Lymphoid, Diffuse”[tiab] OR “Lymphoma, Histiocytic, Diffuse”[tiab] OR “Lymphoma, Large Cell, Diffuse”[tiab] OR “Lymphoma, Diffuse Large-Cell”[tiab] OR “Lymphoma, Diffuse Large Cell”[tiab] OR “Diffuse Large-Cell Lymphoma”[tiab] OR “Diffuse Large Cell Lymphoma”[tiab] OR “Diffuse Large-Cell Lymphomas”[tiab] OR “Diffuse, Large B-Cell, Lymphoma”[tiab] OR “Histiocytic Lymphoma”[tiab] OR “Histiocytic Lymphoma, Diffuse”[tiab] OR “Diffuse Histiocytic Lymphoma”[tiab] OR “Diffuse Histiocytic Lymphomas”[tiab] OR “Lymphoma, Diffuse Histiocytic”[tiab] OR “Large Lymphoid Lymphoma, Diffuse”[tiab] OR “Large-Cell Lymphoma, Diffuse”[tiab] OR “Large Cell Lymphoma, Diffuse”[tiab] OR “Diffuse Large B-Cell Lymphoma”[tiab] OR “Diffuse Large B Cell Lymphoma”[tiab] OR “Lymphoma, Large-Cell, Diffuse”[tiab])

AND

(“MicroRNAs”[MeSH] OR “MicroRNA*”[tiab] OR “miRNAs”[tiab] OR “Micro RNA”[tiab] OR “RNA, Micro”[tiab] OR “miRNA”[tiab] OR “Primary MicroRNA”[tiab] OR “MicroRNA, Primary”[tiab] OR “Primary miRNA”[tiab] OR “miRNA, Primary”[tiab] OR “pri-miRNA”[tiab] OR “pri miRNA”[tiab] OR “RNA, Small Temporal”[tiab] OR “Temporal RNA, Small”[tiab] OR “stRNA”[tiab] OR “Small Temporal RNA”[tiab] OR “pre-miRNA”[tiab] OR “pre miRNA”[tiab])

**ISI:**

TS=(“Lymphoma, Large B-Cell, Diffuse” OR “Lymphoma, Histiocytic” OR “Histiocytic Lymphomas” OR “Lymphoma, Large Lymphoid, Diffuse” OR “Lymphoma, Histiocytic, Diffuse” OR “Lymphoma, Large Cell, Diffuse” OR “Lymphoma, Diffuse Large-Cell” OR “Lymphoma, Diffuse Large Cell” OR “Diffuse Large-Cell Lymphoma” OR “Diffuse Large Cell Lymphoma” OR “Diffuse Large-Cell Lymphomas” OR “Diffuse, Large B-Cell, Lymphoma” OR “Histiocytic Lymphoma” OR “Histiocytic Lymphoma, Diffuse” OR “Diffuse Histiocytic Lymphoma” OR “Diffuse Histiocytic Lymphomas” OR “Lymphoma, Diffuse Histiocytic” OR “Large Lymphoid Lymphoma, Diffuse” OR “Large-Cell Lymphoma, Diffuse” OR “Large Cell Lymphoma, Diffuse” OR “Diffuse Large B-Cell Lymphoma” OR “Diffuse Large B Cell Lymphoma” OR “Lymphoma, Large-Cell, Diffuse”)

AND

TS=(“MicroRNA*” OR “miRNAs” OR “Micro RNA” OR “RNA, Micro” OR “miRNA” OR “Primary MicroRNA” OR “MicroRNA, Primary” OR “Primary miRNA” OR “miRNA, Primary” OR “pri-miRNA” OR “pri miRNA” OR “RNA, Small Temporal” OR “Temporal RNA, Small” OR “stRNA” OR “Small Temporal RNA” OR “pre-miRNA” OR “pre miRNA”)

**Scopus:**

TITLE-ABS-KEY(“Lymphoma, Histiocytic” OR “Histiocytic Lymphomas” OR “Lymphoma, Large Lymphoid, Diffuse” OR “Lymphoma, Histiocytic, Diffuse” OR “Lymphoma, Large Cell, Diffuse” OR “Lymphoma, Diffuse Large-Cell” OR “Lymphoma, Diffuse Large Cell” OR “Diffuse Large-Cell Lymphoma” OR “Diffuse Large Cell Lymphoma” OR “Diffuse Large-Cell Lymphomas” OR “Diffuse, Large B-Cell, Lymphoma” OR “Histiocytic Lymphoma” OR “Histiocytic Lymphoma, Diffuse” OR “Diffuse Histiocytic Lymphoma” OR “Diffuse Histiocytic Lymphomas” OR “Lymphoma, Diffuse Histiocytic” OR “Large Lymphoid Lymphoma, Diffuse” OR “Large-Cell Lymphoma, Diffuse” OR “Large Cell Lymphoma, Diffuse” OR “Diffuse Large B-Cell Lymphoma” OR “Diffuse Large B Cell Lymphoma” OR “Lymphoma, Large-Cell, Diffuse”)

AND

TITLE-ABS-KEY(“MicroRNA*” OR “miRNAs” OR “Micro RNA” OR “RNA, Micro” OR “miRNA” OR “Primary MicroRNA” OR “MicroRNA, Primary” OR “Primary miRNA” OR “miRNA, Primary” OR “pri-miRNA” OR “pri miRNA” OR “RNA, Small Temporal” OR “Temporal RNA, Small” OR “stRNA” OR “Small Temporal RNA” OR “pre-miRNA” OR “pre miRNA”)

**Embase:**

(“Lymphoma, Histiocytic”:ti,ab,kw OR “Histiocytic Lymphomas”:ti,ab,kw OR “Lymphoma, Large Lymphoid, Diffuse”:ti,ab,kw OR “Lymphoma, Histiocytic, Diffuse”:ti,ab,kw OR “Lymphoma, Large Cell, Diffuse”:ti,ab,kw OR “Lymphoma, Diffuse Large-Cell”:ti,ab,kw OR “Lymphoma, Diffuse Large Cell”:ti,ab,kw OR “Diffuse Large-Cell Lymphoma”:ti,ab,kw OR “Diffuse Large Cell Lymphoma”:ti,ab,kw OR “Diffuse Large-Cell Lymphomas”:ti,ab,kw OR “Diffuse, Large B-Cell, Lymphoma”:ti,ab,kw OR “Histiocytic Lymphoma”:ti,ab,kw OR “Histiocytic Lymphoma, Diffuse”:ti,ab,kw OR “Diffuse Histiocytic Lymphoma”:ti,ab,kw OR “Diffuse Histiocytic Lymphomas”:ti,ab,kw OR “Lymphoma, Diffuse Histiocytic”:ti,ab,kw OR “Large Lymphoid Lymphoma, Diffuse”:ti,ab,kw OR “Large-Cell Lymphoma, Diffuse”:ti,ab,kw OR “Large Cell Lymphoma, Diffuse”:ti,ab,kw OR “Diffuse Large B-Cell Lymphoma”:ti,ab,kw OR “Diffuse Large B Cell Lymphoma”:ti,ab,kw OR “Lymphoma, Large-Cell, Diffuse”:ti,ab,kw)

AND

(“MicroRNA*”:ti,ab,kw OR “miRNAs”:ti,ab,kw OR “Micro RNA”:ti,ab,kw OR “RNA, Micro”:ti,ab,kw OR “miRNA”:ti,ab,kw OR “Primary MicroRNA”:ti,ab,kw OR “MicroRNA, Primary”:ti,ab,kw OR “Primary miRNA”:ti,ab,kw OR “miRNA, Primary”:ti,ab,kw OR “pri-miRNA”:ti,ab,kw OR “pri miRNA”:ti,ab,kw OR “RNA, Small Temporal”:ti,ab,kw OR “Temporal RNA, Small”:ti,ab,kw OR “stRNA”:ti,ab,kw OR “Small Temporal RNA”:ti,ab,kw OR “pre-miRNA”:ti,ab,kw OR “pre miRNA”:ti,ab,kw)
